# Analectic

**Published:** 1837

**Authors:** 


### MISCELLANEOUS INTELLIGENCE.

### ANALECTIC, ANALYTICAL, AND ORIGINAL.

### 1.—ANALECTIC.

#### AD SYLVAS NUNCIUS.

1. *Epidemic Influenza.*—This affection, which we noticed in our preceding No. as having prevailed last winter in certain parts of Europe, seems to have had a very extensive range. It is said to have made its appearance at Sidney, New South Wales, early in October, 1836, and at the Cape of Good Hope on the first of the succeeding month. It broke out in the northren parts of Scotland before the end of the year, and was prevalent somewhat earlier in the counties bordering on the Baltic; so that its occurrence there and at New South Wales was contemporaneous.

Our readers are in possession of pretty full descriptions of the epidemic as it appeared in England, and we shall now lay before them the best accounts of it that have reached us of the phenomena it presented in France, Belgium and Denmark.—*Am. Jour. Med. Sciences.*

2. *Influenza in France.*—*La Lancette Francaise*, of the 28th of February last, contains a highly interesting lecture by M. Andral, on the epidemic, as it appeared in France, from which we extract the following observations:

The disease occurred at all ages, and in both sexes. There were few cases, however, in children under two years; a large number of twelve years were attacked, but the greater number of those affected were adults; old persons were not exempted from it; it has been observed in individuals of 85 years of age. It occurred among all ranks and conditions of society, and in every locality. It was not observed to be more prevalent or severe in populous or crowded situations, or where the rules of hygiene are less observed than in those quarters of the city inhabited by the rich. In one house sometimes a single individual was affected, all the rest escaping the disease; this, however, was very rarely the case. In other dwellings, all the inhabitants have been attacked, either simultaneously or successively. A very striking feature of the influenza is a certain disturbance of ennervation, especially of sensibility and motility, and more rarely of the intellect. From the very onset there is pain of the head, which oppresses the patient, and almost prevents him from moving the part. It is chiefly felt about the centre of the forehead, and is of a lancinating character, and accompanied with a sensation of heat or of extreme pressure, with a sense of stupor and vertigo. Some patients are so much affected by it, as to say if it continues they shall perish of a brain fever or an attack of apoplexy. The face at the same time is red, the eyes injected and watery; the ears are the seat of a distressing humming sound. Some patients complain of a prickling in different parts of the surface, especially of the palms of the hands, which in many cases present nothing remarkable to the sight or touch, while in others they are red, swollen, and erythematous, particularly towards the junction of the last phalanges with the metacarpus. In others the pain was less superficial, darting through the substance, as it were, of the limbs; in others the pains were of a contusive character, and principally felt about the larger joints. The pains in general were experienced from the onset of the disease. The pain of the head and stiffness of the limbs, were in many cases the only symptoms that the patients experienced; in other cases, there was in addition, a sense of contraction of the chest, preventing a full dilatation of its parietes, and threatening suffocation; the latter phenomena did not assume, in any case, a very alarming aspect.

In almost every instance there took place a sudden depression of the muscular strength, which continued often during the whole course of the disease, and persisted even after the disappearance of the other symptoms. Very painful and distressing cramps occurred in some cases, but happily in very few. The intellect, excepting in very few instances, presented nothing remarkable; the patients were, it is true, greatly depressed, but otherwise their mental faculties were in no degree impaired. In some who suffered from intense febrile excitement a temporary delirium was observed. Sleeplessness was in a great number of cases very obstinate.

In the greater number of cases the tongue was broad, humid, and covered with a whitish coating; in some, however, it was red and dry. The posterior part of the fauces presented nothing remarkable. The pain in the throat of which the patient complained had not its seat in the pharynx, but in the air passages. Declutition was always easy. The appetite failed during the febrile stage, and this inappitency often continued after the removal of the other morbid phenomena. There was in general but slight thirst. Some patients experienced a sense of weight at the epigastrium, but rarely intense pain. The abdomen has remained, in by far the greater number of cases, supple and without tenderness. The evacuations by stool were not natural; constipation was a more frequent symptom than diarrhœa. In some cases, either as an effect of the disease itself or of its complications, a very obstinate vomiting and a copious diarrhœa, with colicky pains, were observed. This was rarely the case at Paris, but at Passy it appears to have been more common.

The mucous membrane of the nares was in a great number of instances the seat of a mucous flux and of hemorrhages. The latter were frequently slight, but in some cases sufficiently profuse to require the plugging up of the nasal fossæ. The voice was almost always altered. More or less hoarseness, with pain along the course of the larynx and trachea, were characteristic symptoms of the epidemic. Cough was rarely absent; the attack frequently commenced with it; it was very obstinate in some patients, and persisted for a long time after the disappearance of the other symptoms. It frequently occurred in paroxysms, which were very painful and fatiguing. It was sometimes dry, and at others accompanied with an expectoration of a gluey, whitish and transparent matter, containing globules of air. In some cases the sputa were more consistent and opaque, and resembled those we observe towards the termination of an acute bronchitis. In such cases the cough continued for a longer period; it was occasionally accompanied with a sense of strangulation so intense as to seem to threaten asphyxia. The cough had its origin in the larynx and trachea, and rarely proceeded from the bronchi. Thus, in by far the greater number of instances, ausculation and percussion of the thorax gave only negative indications. In some cases, nevertheless, an engorgement and thickening of the mucous membrane was shown to exist by a dry and sonorous rhonchus.

Sometimes the respiration was normal; at others, there was a great dyspnœa. In some cases the impediment to respiration arose from a very acute pain in the parietes of the chest, which prevented its dilatation. When the dyspnœa was severe, there took place an alteration of the features to a greater or less extent, with violet tint of the face and coldness of the extremities. But these symptoms were soon removed by energetic treatment. Death never resulted from them. The oppression of the chest increased considerably where a secondary inflammation of the pleura and of the lungs has manifested itself. It is proper to notice as another cause of dyspnœa, the pseudo-membranous inflammation of the bronchii pointed out by M. Nonat. This is by no means rare, and it would be more commonly detected if greater care were taken to examine the final ramifications of the bronchii. In old persons a very profuse secretion took place from the bronchial mucous membrane, which could not be expelled by the cough, and gave to the case all the characteristics of catarrhus suffocativus.

In a few instances the influenza was unattended with fever; but in the greater number of cases the skin was hot, occasionally dry, but more frequently moist; the pulse was frequent, developed and rebounding; the action of the heart being strong and energetic. The fever continued from two to five days; it rarely lasted beyond the latter period; when it shows itself after the fifth day, we should suspect an inflammation of the bronchii or some other portion of the respiratory apparatus. In some patients a singular tendency to lypothemia and syncope was observed. The perspiration was in some cases very abundant; it commenced with the fever, continued during its presence, and persisted after its cessation. The abundance of the perspiration recalled to our memories the *sweating sickness*. Sometimes the surface presented only a simple moisture; in a certain number of cases a very copious discharge from the skin occurred about the second or third day, at the period when the febrile excitement ceased. The perspiration was occasionally accompanied with a miliary eruption.

We may distinguish in the course of the influenza three periods. The first, which is seldom wanting, is characterized by cough, pain in the throat, cephalalgia, and contusive pains in the limbs. Cough and pain in the throat, of themselves, do not constitute a real case of the disease; there must exist at the same time the several nervous symptoms already referred to. In difficult cases, nervous, thoraric or abdominal symptoms may predominate. The duration of the first period is from one to two days; when it is wanting the disease commences with the second or febrile period. This is characterized by fever, accompanied with most of the symptoms which we have described as its concomitants. This period succeeds often in an insensible manner to the former, but more generally it occurs suddenly, either subsequent to, or without being preceded by the first. The cephalalgia augments as well as the debility and prostration; some patients are struck down, as it were, by lightning, either while walking or attending to their usual occupations. This period continues for about the same period as the fever of measles or scarlatina.

The third period, called apyretic, is distinguished by the cessation of fever, but generally certain of the morbid phenomena of the proceeding periods persist; these phenomena have especial reference to the functions of the central thoracic and abdominal organs. Thus we have pain of the head, and depression; and a cough, which continues, in general, for a considerable period. Although in the preceding stages the stomach presented no indications of disease, we find not frequently that its functions are disordered. The tongue is covered with a thick coating; the mouth is pasty and bitter; the thirst is considerable. If the patients partake of food, it appears to them to have a disagreeable taste; after its ingestion the stomach swells and becomes the seat of a disagreeable sense of weight; with these symptoms constipation of the bowels is usually conjoined. The duration of the third period is very variable; it sometimes terminates at the end of two or three days, at others it continues for a longer or shorter period.

In Paris, during the epidemic of the present spring, the influenza terminated favorably in the great majority of cases. Where death occurred, it was the result of complications, such as pneumonia, general bronchitis, &c. It was, however, a very serious disease when it attacked those laboring under chronic inflammation of the chest.

Dissection has not discovered in the bodies of those who died of the disease, other lesions than those referable to the disease with which the influenza was complicated.

M. Andral does not consider the influenza to be either a laryngitis, tracheitis, or pulmonary catarrh; either of these affections may occur in patients laboring under the disease, and constitute one of the principal elements of the disease, but they do not constitute of themselves the influenza. This is a general disease, the nature and course of which, as in most of the epidemic affections which occur at variable intervals, are both unknown.

The treatment will vary according to the symptoms. When indications of cerebral congestion, with more or less febrile excitement present themselves, we should not hesitate to open a vein, and at the same time apply revulsives to the extremities. If the fever is moderate, the pain in the head inconsiderable, and the oppression slight, rest and dilutent drinks will be sufficient. If the mouth is pasty or bitter, the tongue foul, and more or less aversion from food is present, with a sensation of weight at the epigastrium, emetics 18 to 24 grains ipecac. or two of tartar emetic may be given with great advantage. If the cough is dry, painful and fatiguing, narcotics, as belladona and opium, should be prescribed.

If in the second period any particular symptom manifests itself, we should insist upon an attention to regimen, and the use of mucilaginous drinks. In the third period, if the symptoms of stomachic or intestinal disorder predominate, vomits, or still better, purgatives will succeed in removing it. During convalescence, it has been found necessary to restore tone to the stomach by the use of bitters and tonics.

*Ibid.*

---

3. *Debate in the Academy of Medicine of France relative to the Influenza.* February 14, 1837.—M. Lepelletier de la Sarthe, having charge of the *Bureau Central*, and supplying the place of one of the physicians of the *Hotel-Dieu*, who is sick, has had an opportunity of treating a large number of patients afflicted with the epidemic: the number in twenty days amounted to 1050. Besides its occult cause, the epidemic principle, M. L. recognises also as causes of the influenza, atmospherical variations, and particularly cold combined with moisture. He conceives the disease to depend essentially upon an inflammation of the bronchial mucous membrane, but distinguished by a certain nervous affection—constituting a spasmodic bronchitis. The disease may assume various forms, but it is easy to discover in all cases the same leading concourse of symptoms. Of itself, the influenza is always a benign disease; when more serious symptoms develope themselves, these are referable to some complication. In two hundred cases of influenza, M. L. observed twenty-five of pneumonia, two of pleurisy, three of gastro-entaritis, two of acute rheumatism, and two of parotiditis. He has seen a phthisical patient suffocated upon the attack of spasmodic bronchitis, and die asphyxiated—the same has occurred in many old persons laboring under catarrh. The influenza may assume a very serious character in apoplectic subjects, which is explained as well from the cerebral congestion caused by the cough as from the prescription, as it were, of blood-letting in this disease. The complication the most frequently fatal is pneumonia or pleurisy, more especially as blood-letting, although strongly indicated, in such cases, has not the same advantageous effects as under other circumstances. M. L. has found the use of emetic tartar, in large doses with bleeding, to be the most efficacious treatment in such cases. He has also found advantage to result in old persons affected with catarrh from the employment of the white oxyde of antimony. M. L. was struck with the tolerance exhibited for the tartar emetic; of eighteen patients to whom it was given in large doses, two only vomited.

M. Louyer Villermay also regards the iufluenza as a slight disease. He does not join in the opposition which some physicians evince to the use of blood-letting, from which he has obtained great advantage whenever the pulse was full and developed, and the respiration oppressed, &c. The disease was in that manner cured in three days; the blood presented a firm coagulum, and often a thick buffy coat.

M. Recamier referred to the epidemic of 1803, which was very fatal. The course of the disease was then most frequently by a cutaneous inflammation. M. R. regards the influenza as an affection of the nature of the eruptive fevers. This opinion is not founded merely upon the phenomena of the epidemic of 1803, but upon the concourse of symptoms which are analagous to the two classes of disease. The catarrh common to influenza is in effect the *cattarrhe tupiculeux* of scarlatina; and if in 1837 the cutaneous eruption was not general, very often an erysipelatous redness was observed, and pustules of the lips were invariably present. Finally, it is known that in the eruptive fevers, the eruption does not always occur, but that the *nature* of the disease is not in consequence changed. The eruption, besides, may take place in the interior; this was the case in 1803, when the digestive mucous membrane, as well as the exterior of the body, was the seat of the eruption. The internal eruption had all the characteristics of the lesion so well described by Rœderer and Wagler. Whenever an epidemic prevails with any degree of severity, it always leaves after it indelible traces; thus since the epidemic of 1803, M. R. has seen an increase of the intestinal eruptions. M. Recamier distinguishes in the phenomena of influenza the leading forms: in the first, the inflammatory form, the persons affected are generally strong and robust, with a hard resisting pulse, there is acute pain of the head, difficult respiration, and the base of the chest being, as it were, bound with a cord. In this form bleeding is loudly demanded. M. R. has repeated it from four to six times, and he has seen the blood become more and more coagulable in proportion as the bleedings were repeated, a character different from what is observed in pleurisy and pneumonia, and which seems peculiar to influenza. In the second form, the bilious, there is a bitter taste in the mouth, the tongue is white, pasty, and covered with a mucous coating; the pulse has neither form nor resistance; the disease principally affects the digestive organs. Here emetics are indicated and their effects are immediate. M. R. has seen all the symptoms disappear in twenty-four hours under this treatment. Purgatives are much less efficacious, which is easily understood, inasmuch as the emetic, besides the evacuation it produces, causes also diaphoresis, which is the genuine crisis of the disorder. The third form of influenza M. R. terms the nervous. There is here extreme nervous excitement, loss of sleep, transient pains of the limbs and trunk, the pulse is small and depressed, and the prostration of the patient considerable. In such cases bleeding is positively forbidden. M. R. has seen it under such circumstances increase the intensity of pneumonia. Under this form the influenza is a very grave disease; the patients often sink before any means can be employed to excite reaction. What M. R. has found most successful in such cases is the use of baths.

M. Piorry recognises different phases in the epidemic. He has observed the pulmonary inflammation to extend deeper and deeper, confined at first to the first divisions of the bronchii, it has subsequently reached its final ramifications, which is the grade of the disease now prevailing. M. P. has used, without success, the tart. ant. in large doses, in the pneumonia accompanying the influenza, especially in old persons. He has not been more successful in the use of blood-letting, even when repeated at short intervals.

M. Bouillaud, without denying a special epidemic cause giving rise to influenza, discovers in the atmospheric constitution a sufficient explanation of its production and propagation. He does not deny but that the epidemical diseases in general stamp upon all intercurrent diseases their own peculiar characteristics; nevertheless, he believes that this proposition has been exaggerated. He has not as yet collected a sufficient number of facts to be able to pronounce with certainty upon the particular character ascribed to the pneumonia accompanying the influenza, but perhaps sufficient to show that repeated bleedings have not the same beneficial effect as in common pneumonia. M. B. cited especially the case of a physician 68 years old, in whom bleeding repeated at short intervals quickly cured a pneumonia of a most serious character. M. B. observed that since the invasion of the epidemic more patients had died under his care than there had during the preceding eight months.—*Ibid.*

---

4. *Influenza in France.*—We derive the following remarks on this subject from the *Gazette Medicale de Paris*, of February 18th, 1837. "All our practitioners have remarked, that during the last ten days, the epidemic has deviated from its original character, either in consequence of an increase in its intensity or the occurrence of concomitant diseases. Thus, instead of a slight bronchial catarrh presenting the same concourse of symptoms in all the individuals attacked, we now observe both the original form of the disease, but accompanied by pneumonia of a general character of much greater intensity, and pneumonia of a very severe and invidious grade, accompanied with a tendency to prostration, while in other cases the pneumonia of the disease indicate especially disturbance of the digestive organs, or of the nervous system, with a morbid condition more or less alarming, of the whole organism. In place of terminating in a few days with the aid of simple remedies, the disease now requires prompt and active treatment. Bleeding, purgatives, emetics, large doses of tartarized antimony fail often in preventing a fatal termination. The mortality of the disease is, in fact, much more considerable than formerly. M. Bouillaud has lost during the last eight days in his clinical practice, a greater number of patients affected with it than during the preceding eight months.

Are the few pathological forms under which the influenza now presents itself, to be considered as independent diseases, as many members of the Academy believe them to be, or should we attribute the increase in its mortality within the last few days to its being complicated with other more serious affections? This is a very important question, upon the proper solution of which will depend, in a great measure, the plan of treatment it is proper to pursue. We believe with M. Recamier and many other distinguished physicians, that the increased mortality among the sick is caused by the epidemic alone; and this opinion rests upon the following leading considerations:

Almost all physicians have observed that the influenza manifests itself at first by a concourse of general symptoms which affect the respiratory operation only. Secondarily. The occurrence, in the greater number of instances, of the bronchial form of the disease, has led many to suppose that the pneumonic form was a different or complicated disease. This error results from the supposition recently entertained by some pathologists, that diseases differ decidedly from each other according as they are seated in different parts of the same apparatus. M. Recamier very properly considers the reigning disease to be an eruptive fever, the pneumonia and anatomical results of which may occupy any portion of the skin, the presence of the disease not being evidenced merely, however, by its exterior manifestations. An epidemic is a general morbific cause of a specific character, which affects an entire population, and which, when once introduced into the organism, gives rise to symptomatic phenomena variable in character, but referable to one and the same cause. It is the same in relation to an epidemic cause of disease as it is in relation to any specific cause of disease, as scrofula and rheumatism, and particularly morbific causes of a more material nature, as the emanations of lead. Should we consider all the pathological manifestations dependent upon these morbific causes as independent diseases, because they present themselves under different forms or attended with different anatomical symptoms?

It has been already remarked, that this question has a powerful influence upon the treatment of the epidemic. If the local phenomena it presents be in effect dependent upon one and the same morbific cause, it is important that they should all be submitted to a general plan of treatment, adapted to their nature, and modified only so far as individual indications may demand. Thus we find the influenza attended in different cases with an affection of the throat, of the bronchia, of the lungs, with gastro-enteritis, or with more general symptoms. In all these cases it is the same disease, or in other words, these different cases present various phenomenal or symptomatic indications of the same morbid condition, or of the effects of a similar cause; and the general treatment adapted to remove this cause should be the same in all, with such variations only as are necessary to fulfil secondary indications.

At the last session of the Academy, several distinguished practitioners detailed their experience in relation to the proper treatment of the epidemic. M. Lepelletier, of Mans, believed that he had observed blood-letting in place of being salutary, to have a tendency to sink the patients into a state of collapse; he gives the preference to large doses of emetic tartar in the most severe cases. To this treatment eighteen out of twenty cases of the pneumonic form of influenza yielded rapidly. He likewise observed, that in such cases tolerance of the emetic tartar was readily established. M. Recamier has had recourse to a mixed treatment, sometimes depletive, at others evacuent, and at others narcotic, according as certain indications presented themselves, resulting from the difference of the morbid phenomena in different cases. MM. Louyer Villermay and Bouillaud appear to have derived advantage from blood-letting. They have not, however, presented results of their practice of so explicit and positive a character as either of the preceding gentlemen. M. Recamier has in fact declared that the cases of pneumonic influenza treated by blood-letting repeated on several successive days, but not repeated several times on the same day, have proved fatal.

When the symptoms of influenza are slight, it is well known that the patient readily recovers with very simple remedies, or even none; but when of a severe, aggravated character, or when the parenchyma of the lungs becomes affected it requires for its removal an active and well directed treatment. The advocates of bleeding have themselves remarked that this remedy does not appear during the prevalence of the epidemic, to produce the same good effects it does under ordinary circumstances. It is difficult to say precisely to what extent bleeding is either injurious or beneficial in influenza, or what are the remedies which should be preferred to it. When the disease is slight it will disappear under almost any treatment; but notwithstanding it may in no case terminate fatally, yet recovery may be more or less rapid and complete according as it is submitted to a treatment more or less judicious. In the commencement of the epidemic the disease was left pretty much to itself, but it being remarked, as it was supposed, that the patients thus treated remained generally in a state of more or less debility, with anorexia of longer or shorter continuance, purgatives were subsequently very generally employed, with the effect of dissipating these remnants of the disease. When administered twenty-four to thirty-six hours after the invasion of the malady, the disease was found to be shortened in its duration, to lose speedily its general symptoms, and to be prevented from changing into a serious affection. Of seventy or eighty patients treated in this manner, all recovered without any dangerous or consecutive symptom. Many of the patients in whom the disease was left to itself at its commencement, were observed to become affected with more serious symptoms, while those who were bled on the first attack did not recover, or very slowly."

---

5. *Influenza in Belgium.*—According to M. Gouzee, the epidemic, as it occurred at Antwerp during the last winter, was of a much more serious character than that which prevailed in either 1831 or 1833. He remarks that the disease was not a simple pulmonary catarrh, modified by the epidemic constitution—it was not an inflammation of the bronchial mucous membrane, notwithstanding a cough was most generally one of the predominant symptoms. The cough when it existed, for it was occasionally absent, had in fact peculiar characters, not common to the cough of catarrh. It was small, superficial, if such an expression may be made use of, occurring without any effort, and fatiguing only by its continuance or the extreme irritability of the patient. It not unfrequently occurred in paroxysms, particularly towards the evening or in the morning; being sometimes dry, and at others accompanied by an expectoration of mucous mixed with serum. Exploration of the chest exhibits no impediment to the free expansion of the lungs. If to the above character we add that the cough is almost instantaneously relieved, as M. G. has frequently observed, by the employment of sedatives,—recollecting that under any circumstances, it is to be viewed as a symptom of little importance, and that it is often entirely absent, we have a right to conclude that it does not depend upon an inflammation of the bronchial mucous membrane, the existence of which is evidenced by no other phenomena of the disease.

Certain symptoms, which occur very generally, such as coryza, pain at the upper part of the larynx, and hoarseness, seem to indicate that the cough may depend upon an irritation of the larynx; but the superficial and fugitive alteration with which those symptoms are connected, appears to M. G. to be merely accessary and not sufficient to explain the other predominant phenomena which give to influenza its peculiar character. These phenomena he believes to result from a disturbance of innervation, upon which, he is of opinion, the disease chiefly depends.

This disturbance of innervation are, he remarks, strikingly evinced by the phenomena of influenza. Pain of the head is constantly experienced in the commencement especially; it is then frequently confounded with a sense of fulness in the nostrils, extending to the eyes and ears; it is attended with vertigo, with a sort of pressure over the eyes, a singular sensibility of the integuments of the head, sleeplessness, and many times slight temporary delirium during the night. The patients are weak, suffer from an oppression of the chest, pains in the back, loins, and extremities; they are quickly exhausted, and liable even to attacks of complete syncope. To the foregoing symptoms, which are purely nervous, are added, in the commencement of the attack, symptoms of a more or less decided reaction. To these M. G. has frequently to present an exacerbation in the morning, and a remission towards evening. The face is red; the skin hot and moist; the pulse moderately frequent, and, as M. G. has always observed, remarkably soft. He has seen some patients who were young and robust, affected with a profuse epistaxis, which produced but little abatement of the disease. The symptoms of reaction disappear by the second or third day, but a sense of debility persists, which bears no relation to the slight degree and short duration of the proceeding symptom. The patients continue to be affected with a lightness of the head, a sense of fatigue, pain, sleeplessness, and return, for a longer or shorter time, of their slight cough, which causes in the thorax a peculiar sensation of fatigue.

The disturbances observed in the digestive organs, the same as those of the organs of respiration, are equally referable to a derangement of innervation. To anorexia and a distaste for animal substances is often joined distressing nausea, vomiting, a sensation of dryness in the mouth, and thirst, nevertheless, the tongue is expanded, moist, and covered only with a slight whitish coating. The pain of the throat, the difficulty of deglutition, have their seat in the larynx. M. G. has never found the tonsils swollen. Frequently a slight degree of redness exists at the posterior part of the fauces. The bowels are variable—generally they are costive; but diarrhœa occurred in some cases towards the close of the epidemic.

Many patients complained of an acute pain at the epigastrium and in the hypochondria. We must be cautious not to take these pains for the indications of a gastritis. They are the simple result of the fatigue caused by the cough, which is the more sensibly felt according as the patient is the more weak and irritable; the pain ceases with the cough.

When first attacked with influenza, the patients seem to be threatened with a serious disease, nevertheless we need seldom hesitate to promise him a favorable issue. The patient is seized either suddenly, or after having for a few days experienced irregular chills or a more increased susceptibility to cold; but however alarming the symptoms are at the onset, they abate by the second or third day, leaving the patient merely affected with a slight cough, which now often becomes more dry; he remains in a state of feebleness, is easily fatigued, experiences vertigo and a painful sense of pressure over the eyes. But this condition of things has nothing in it to excite inquietude, if we except its remarkably long continuance.

In the commencement of the epidemic, the violence of cough and the phenomena of reaction induced M. G. to employ leeches, to the extent of ten or fifteen, applied beneath the clavicles, or of six to eight upon the larynx; but in no case did they cause an amelioration of the symptoms. The only cases in which venesection was resorted to, were those in which there occurred symptoms of bronchitis, pleuritis or pneumonia, and which generally terminated fatally. The treatment was generally confined to the administration of mild and tepid drinks, with confinement to bed in severe cases, pediluvia and diet, to which treatment the phenomena, the most serious in appearance, have promptly ceded. The patients who consented to remain in bed a few days, were much more promptly and certainly cured, than those who merely kept their chamber. M. G. has observed some relapses, these have not been so dangerous as has been asserted; the patients, however, were longer in acquiring their full strength.

The cough which frequently continues during convalescence, becomes a serious symptom in weak and delicate patients. M. G. has seen it, in nervous females, recur in paroxysms of many hours duration, of so violent and painful a character, that the breast appeared to be, as it were, torn, while the patients were sunk into a state of extreme exhaustion. Some large tea-spoonful of the syrup of lactutcarium, properly prepared, taken as soon as the coughing commenced, have always speedily diminished its violence and caused it gradually to abate. M. G. has also employed in such cases, the powdered belladona, mixed with white sugar, in the dose of a grain, taken about the period when the cough is expected to return, without exceeding, however, two or three grains a day, and in many cases this has removed the cough as it were by enchantment. The water of the cherry laurel, the syrup of the white poppy, and opium, he has not found so efficacious. Strengthening aliments, and some degree of exercise, are very proper towards the termination of the disease.

Influenza is not a dangerous disease, excepting when complicated with acute affections of the chest; it may, however, cause diseases of a very prompt and serious nature to become developed in persons predisposed to affection of the chest. It was found to augment in a very decided degree, especially towards the close of the epidemic, the mortality in the Military Hospital and city of Anvers.

Very little disease prevailed during the whole of the year 1836, which was the case also previous to the occurrence of the cholera in 1832. The autumn had been very wet. The first cases of the influenza occurred at Antwerp, about the middle of January, 1837. Females, delicate and nervous persons, in easy circumstances, were the first attacked. The disease soon spread; by the commencement of February nearly the whole population was attacked. By the 15th to the 20th of the month, new cases became more and more rare, and about this period there took place a manifest decrease in the disease.

M. G. has seen the influenza in persons of all ages, excepting children under one year. The disease did not exhibit any degree of intensity when it occurred in infants, it was chiefly confined to a slight cough, coryza, running of the eyes, a fever of one day's continuance, and a few days of debility. He has seen it in adults, complicated with intense bronchitis, pleurisy, pneumonia, pericarditis, pulmonary emphysema, and irritation of the brain, with continued delirium; many of these cases terminated fatally.

A fact worthy of notice was remarked at the Military Hospital; that intermittent fever, previously of tolerable frequency, ceased to present itself during the continuance of the influenza.—*Bulletin Med. Belge.*, February, 1837.

---

6. *Influenza at Copenhagen.*—From an account by Professor Otto, of Copenhagen, of the influenza as it appeared in that city, contained in the *Zeitschrift fur die gesammte Medicin*, for June, 1837, we extract the following particulars:

The disease made its appearance in Copenhagen about the middle of December, 1836. The epidemic occurred suddenly, and soon after its appearance, simultaneously in St. Petersburg, Stockholm, Berlin, &c. As in all former similar epidemics, it was preceded for a long period by damp and cloudy weather, which also continued during the whole time the disease prevailed: unlike the former epidemics, it commenced in the beginning of winter, while they generally occurred in spring. In December, 1835, which was likewise damp and cloudy, there prevailed an epidemic of measles. The influenza spread throughout the city with uncommon rapidity; one family after another became attacked with it in quick succession, not one of those to whom Dr. O. acted as physician escaped it. By the middle of January, 1837, when the epidemic was at its height, it was estimated that thirty thousand individuals were affected with it, though in all probability the number was even greater, as many did not apply for medical aid but followed their usual occupations. No condition, age or sex escaped its attack. Even those who were laboring under other diseases, and were confined to their chamber, or even to bed, were attacked. The epidemic began to diminish towards the end of January; it continued to prevail, however, during the whole of February, and although cases occurred in the beginning of March, they presented less of the peculiar symptoms of influenza, and by the 28th of the month nothing but ordinary catarrh was obseved.

The disease was frequently ushered in by febrile symptoms, but as frequently without; almost always, however, by a very peculiar loss of strength. The most striking symptom at first was a violent dry cough, sometimes accompanied and at others unattended with a pain in the breast, and hoarseness. There was likewise a discharge from the nostrils, mostly thirst, loss of appetite and pain of the head. To these symptoms there succeeded, in most cases, all the ordinary phenomena of fever, though in many instances no febrile symptoms were present. There was generally some pain of the stomach, coated tongue, nausea, bitter taste in the mouth, &c. They who paid no attention to the first symptom of the epidemic, but went about and attended to their business as usual, experienced in general an augmentation of the cough, defluxion from the nostrils, pain of the head, &c., with symptoms of fever, and were finally obliged to take to their beds; the disease in such lasted often for fourteen days, or even longer. If, on the contrary, on the appearance of the disease, the patient went to bed and took some gentle diaphoretic medicine, all the symptoms frequently disappeared within three or four days. Notwithstanding, however, in this manner the more severe symptoms were often removed or greatly diminished; in most cases the cough and expectoration continued for a long time, in some instances for four to six weeks; the convalescency from the disease was in general tedious, and attended with want of appetite, and especially an inexplicable sense of fulness. An imprudent exposure to cold or dampness during convalescency, gave rise to a relapse, with symptoms of great severity and danger.

The prognosis was almost always favorable. Of the disease under ordinary circumstances no one died; but in those who either were laboring previously under some disease of the chest, or were predisposed to such, or who while affected with influenza in consequence of exposure to cold were attacked with inflammation of the chest, the symptoms were often of a very severe character, and in some cases terminated in death. Old persons in general were in greater danger from the disease than the young, and especially when weakened by the long continued cough and expectoration to which it so frequently gave rise. In children the disease was milder and less dangerous generally than in adults.

The treatment required by the influenza was the same as that proper in catarrh. When attended by foulness of the prima viæ the muriate of ammonia was peculiarly advantageous. Bleeding was seldom demanded. Leeches were occasionally resorted to to remove pain of the side. For the long continued cough the Iceland moss was given in many cases, and the debility which remained so long after the disease called for the employment of strengthening remedies and nourishing diet.

Dr. Otto remarks, that the influenza, as it occurred in Copenhagen, appeared to him to exhibit a contagious character. D. F. C.

*Ibid.*

7. *On the Enlargement of the Spleen, after Agues and other Fevers.*—Before we discuss the question of the probable cause of those enlargements of the spleen, which are so often the consequence of intermittent fevers, it may be useful to glance at some of the most curious and best ascertained facts connected with the healthy anatomy of this viscus. To Professors Felici and Panizza of Pavia, the profession is indebted for an admirable monograph on this subject; wherein they have most indisputably demonstrated the analogy of the texture of the spleen, with that of erectile tissues in general, and especially of the corpora cavernosa of the penis and clitoris. The character of this texture may be exhibited, either by repeatedly washing a portion of the organ with pure water, until it is completely freed from all the blood which it contains; or by blowing air along the splenic vein, so as to distend the cells. The latter of these methods will be found to succeed better when the spleen is enlarged, than when it is, from any cause, contracted in its dimensions. In our brief examination of the anatomy, we shall commence from the exterior of the viscus and proceed inwardly. The outer envelope of the spleen is its serous or peritoneal coat; this adheres firmly to the subjacent fibrous tissue, the tunica propria, or proper investing capsule, which binds or confines the whole organ together in one mass, and from whose inner surface are sent off numerous prolongations, some of which dip between the different portions, or lobules, forming the septa, or partitions between these and others, by joining and rejoining with each other, give rise to the cellular, or recticular texture, which pervades the whole viscus. If the spleen be hypertrophied, the cells may be seen with the naked eye; but, in its natural state, the use of a magnifying glass is necessary to discover them.

The tunica propria and all its prolongations are evidently very contractile; for if we cut a fresh spleen into slices, the contraction of the septa causes all the contained fluid to ooze out, and the divided surface then exhibits a granulated appearance, similar to what we observe in a portion of lung, which is in the second stage of peripneumonia.

The arterial distribution of the spleen deserves particular notice. No sooner do the branches of the splenic artery enter the substance of the viscus, than they become so minutely subdivided, that they can no longer be traced. Assolant has proved, by a multitude of experiments, that, if one of the branches of the splenic artery be tied, the portion of the organ, on which the branch is distributed, is inevitably killed; and he infers therefore that there is but a very imperfect anastomosis between the blood vessels of the spleen. This is confirmed by the fact, that if air is blown into one of the arterial branches, it does not pass freely, if at all, into the others. The anatomy of the veins of the spleen is better understood. They commence by extremely minute twigs, which are continuous with those, of which the cellules are formed, and of which they appear to be the mere prolongations; several of these twigs uniting, give rise to larger branches, whose parietes are perforated with numerous apertures, large enough to admit a probe, and which communicate directly with the cellules. These apertures become smaller and less frequent, as the branches approach the large divisions of the splenic vein, which are usually three or four in number, and which meet at the concave edge of the spleen, into one large trunk; this proceeds on to meet the vena portæ. The lymphatic vessels of the spleen are neither numerous, nor very conspicuous. They are discovered much more easily on its surface than within its texture. The nervous supplies are derived partly from the cœliac plexus, and partly from the par vagum.

Without entering upon the labyrinth of theories, as to the uses and functions of the spleen, we may be permitted to say, that it is, at least, probable from the analogy of its structure with that of the recognized erectile tissues, that there is some substance interposed between its vasa sanguifera, *inferentia, and efferentia:* unless we admit M. Andral's opinion, that "la rate n'est qu'un reseau veineux, où la forme celluleuse tend à remplacer la forme vasculaire." Whether, however, the spleen is to be considered as a mere diverticulum to the blood of the abdominal viscera, during certain states, or operations of the system; or whether it has not at the same time more elaborate duties to perform, (as for example being accessary to the formation of bile, or to the production of blood, either directly, or by generating a peculiar sort of lymph, which is afterwards conveyed by the absorbents into the thoraric duck) remains still a problem of most difficult solution. After these short preliminary remarks, we now approach the question, as to the probable cause of the enlargement of the spleen, which, it is well known, are very frequently induced by intermittent fevers.

Some pathologists have attributed these lesions to a merely passive or atonic accumulation and congestion of blood in the veins or cells of the viscus; and they have altogether excluded the idea of any active increase of vascular action. The cause, according to them, is therefore purely mechanical; the normal contractility of the veins, or cells, being supposed to be overcome by the excessive distention of their parietes.

But surely this mode of accounting for any change in the condition of a living organ is not very consistent with what we observe in other parts of the body. What viscus can be alternately overcharged with and drained of blood, without its vital energies being affected, or without some reaction being induced? And again, when we consider the variety of morbid degenerations, to which the spleen is subject, such as, induration, ossification, softening, conversion into a black pultaceous mass, the formation of genuine purulent matter, and the adhesion of its surfaces to the surrounding organs, surely we shall be forced to believe that some other morbid process, besides that of mere congestion of blood, may affect its substance. Moreover, if the engorgements of the spleen, which occur during the course of intermittent fevers, were owing altogether to an excessive accumulation of blood in the vena portæ, we might naturally expect, that some of the other viscera, whose veins terminate in the same trunk with those of the spleen, should be almost always similarly diseased at the same time. Occasionally, indeed, the liver, as well as the spleen, is found enlarged; but this is by no means of constant, nor indeed of very frequent occurrence. Morgagni was of opinion that the liver is oftener contracted than enlarged in such cases; and on the other hand, it is worthy of notice, that in the majority of the examples of diseased liver, recorded and described in Andral's Clinique Medicale, no notice is taken of any co-existent lesions of the spleen; and that, out of the 187 cases mentioned in Lieutaud's Anatomie Medicale, twenty-three of them only were complicated with splenic alterations.

The very obvious inferences from these facts are sanctioned and confirmed by the physiological consideration, that, as the veins of the spleen are so much larger and more numerous than its arteries, it is not easy to understand, how a stasis of the blood, or a passive congestion can take place in them, unless there be some obstruction in the vena portæ, or vena cava, to the centripetal blood. Such a complication may occur, and does, we know, exist in cases of diseased heart; in which there is a most distressing tendency in almost every organ to a morbid accumulation of venous blood. Hence the source of the apoplexies, dropsies, dyspnœas, engorged livers, hæmorrhoids, &c., which are frequently observed in the progress of cardiac cases.

But, although these reasons may be considered satisfactory to shew that enlargements of the spleen do not depend upon a mere passive congestion of the venous blood in its texture, the question, what is the cause, or rationale of the afflux of the blood from the surface of the body, to the internal parts, and more especially to the spleen in agues? now presents itself to our consideration. Cullen's hypothesis is well known to most medical readers: the primary spasm of the peripheral vessels, the contraction of their diameters, and the consequent expulsion of part of their contents, &c., from the surface to the deeper organs are its leading features. The first movement in the morbid action is, according to Cullen, in the vessels of the skin, and the internal congestions are the necessary and almost mechanical effects of the blood being driven from the skin inwards. Dr. Fallot, the author of the present paper, assumes it to be more consistent with the operations of living bodies, that wherever a sudden alteration in the quantity of blood in any part or organ takes place, we may be satisfied that some change in the innervation of that part or organ has already preceded it; and that it is not the result of a merely mechanical afflux from one point to another. "*Ubi stimulus ibi affluxus*," is a physiological maxim, to which there is no exception; and the converse of the proposition is equally true, that where the excitement is defective, there is the afflux of blood diminished.

If we observe what takes place during the re-action of an ague, we shall find that the feeling, or consciousness, to the patient at least, of the hot stage very distinctly precedes the full throbbing pulse, and warmth of the skin, which are the indications to others of its presence. Shall we then make an exception of the spleen, and consider it as nothing but an inert sponge, at one time soaked with blood, and at another time empty, according as other organs have an excess, or deficiency of the circulating fluid? And when all other erectile tissues are uniformly subject to the law that we have already announced, viz. that of being engorged only when they are stimulated, and of becoming flaccid, when the stimulation is diminished or withdrawn, why shall we apply other laws to the spleen, and suppose that it is excepted from the vital action of similar parts? We infer, therefore, that if the spleen becomes congested and enlarged during an intermittent fever, this congestion and enlargement are the effects of an abnormal afflux of blood, occasioned by the pre-existence of an abnormal stimulation in the part. Observe the changes induced in a splenic tumor in the phases of an ague. Soft, and but little painful during the intermission, it becomes distended, hard, and sensitive during the cold stage; but, it is not, until the vascular re-action of the hot stage is fairly established, "quand les mouvemens d'expansion sont en pleine activité," that it attains its maximum of intumescence, resistance and tenderness. When the irritation is slight or temporary, the vascular excitement subsides, and is gradually dissipated entirely, the swelling being no longer appreciable. When it is more severe, or is persistant for a considerable time, the blood is forced into the areolæ, or minute cellules, which then become so gorged and over-distended, that their parietes take on an inflammatory action, and this state is succeeded by one of induration, or by the deposition of purulent matter. Under these circumstances the spleen, from its augmented bulk, is felt projecting below the edges of the false ribs, on the left side.

Supposing now, that the question which we have been considering, namely, whether the enlargements of the spleen after agues are generally the result of an active or a passive congestion of blood in its texture, to be decided according to the views explained above, another of almost equal difficulty immediately presents itself. This active congestion—is it idiopathic and independent of the affection of other viscera; or is it merely sympathetic, and consecutive on some irritation existing elsewhere?

On the one hand, it may be alleged, that the diseases of the spleen, (with the exception, perhaps, of the enlargement now under discussion,) are very rarely isolated, but are usually co-existent with lesions of other organs; and on the other hand, we must recollect that our ignorance of the healthy functions of this viscus precludes us from detecting its morbid deviations, unless these are accompanied by obvious and visible changes of structure. The subject, therefore, of splenic disease must be one full of uncertainty and difficulty. M. Broussais, in treating of the diseases of the liver, has rendered it probable that this important viscus is very seldom primarily or idiopathically affected, unless from an external injury. What is probable of the liver is much more so of its neighbor, the spleen. The frequent co-existence of splenic disease with diseases of the heart deserves the attention of morbid anatomists, and has not received that minute investigation, which the importance of the subject requires. Dr. Kreisig, of Dresden, who has written a good treatise on Die Krankheiten des Harzes, explains the connexion of splenic with cardiac disease, by reference to the obstruction offered to the free return of the blood in the venæ cavæ, and to the consequent congestion, which all the abdominal viscera must experience under such circumstances. This explanation, though satisfactory to a certain extent, cannot well apply to every case of diseased spleen. According to Kreisig himself, alterations of the spleen are frequently co-existent with diseases of the left, as well as of the right, side of the heart; and Testa, and other Italian physicians allege that many cardiac affections are secondary to, and are apparently induced by, morbid states of the spleen; although this viscus may not have become increased in bulk, and cannot therefore be supposed to have excited any mechanically injurious effect on the free circulation of the blood through the heart and large vessels.

Baron Alibert has suggested in his Nosographie Naturelle, that this sympathy between the spleen and heart may be accounted for, by their nervous connexion, through the medium of the par vagum, which he thinks "partage avec le grand sympathique la faculté de produire des sympathies." Without denying the importance of this connexion, we cannot concede to it the exclusive influence, assigned to it by the Baron: else how comes it, that the liver and stomach, which derive part of their nervous supplies from the same source, viz. the nervi vagi, do not as frequently sympathise with diseases of the heart, as the spleen is known to do? Dr. Fallot is, therefore, induced to believe, that it is rather in consequence of it (the spleen) being an accessary or auxiliary instrument or agent of the circulation, and designed by nature to avert, and in some degree to compensate for the accidents that might ensue from any interruption of this vital function, (whether this arises from an excess, or from a defect of activity), that it is so intimately associated with the heart and lungs. This functional association, or sympathy, is not nearly so obvious and appreciable during health, as when either of these organs is involved in disease.

If the heart and lungs become, from any cause, oppressed with venous blood—let us remember that this state occurs during every cold stage of an ague—and in an obstinate case, how frequent must this oppression or congestion have taken place—the spleen must very speedily suffer, in consequence of the column of blood pressing back upon every part of the portal system; and where do you think, asks M. Fallot, "qu'elle doive retentir plutot que dans la rate, unie au cœur par des liens de syhergic pour l'accomplissement de la meme fonction?" Moreover, the great size of the splenic artery, and consequently the large quantity of blood constantly transmitted through it, must, of a necessity predispose the organ to be affected with various morbid states. It is a subject of great pathological interest, to ascertain, what is the relation, as to cause and effect, between enlargements of the spleen, and the very frequently simultaneous occurrence of alterations in the mass of the blood. It is certainly a fact, known to every experienced physician, than when the spleen has been diceased for any length of time, a dyscrasis of the circulating fluids is almost invariably induced. We cannot afford room, at the present time, to enter upon the discussion of this very curious theme: the mere suggestion of it must be sufficient.—*Bulletin Medical Belge.*

—

It may be useful to subjoin a few comments on some of the statements in M. Fallot's paper, and more particularly upon the suggestion made at its close. It is not quite easy to determine, whether the dyscrasis of the circulating fluids is owing to the morbid state of the spleen, or whether the converse of the proposition be true. However this may be, the constitutional symptoms usually associated with chronic enlargements of the spleen are these: general debility, paleness and a deficiency of red blood in the capillaries; the conjunctivæ are pale and bloodless, the scleroticæ are of a pearly blue color, and the complexion is waxen, as in chlorosis.* If the disease has existed for some time, the extremities are apt to be cold; and the skin is pale, dry, shrivelled, and often furfuraceous. Adults do not usually suffer so much from splenic enlargement, as children do. In younger subjects, it is very apt to induce quickly an incurable marasmus; whereas we not unfrequently observe that, with adults, it may exist for a length of time, without being attended with serious disturbance of the constitution. (Does not this difference seem to indicate that this viscus is probably more or less directly connected with the formation of the blood?) Patients affected with vascular engorgements of the spleen are very prone to foul sloughing ulcers, from slight wounds or bruises; and all restorative processes are observed to be very tardily and imperfectly performed. The blood of such patients is always more or less unhealthy; sometimes it coagulates only imperfectly, and no serum is separated: in other cases the cruor is black, soft, and does not assume a florid hue on the surface, from the contact of the air. In short, not a few of the attributes or symptoms of genuine scorbutus are found in many cases of enlarged spleen. There is a tendency to hæmorrhage from slight wounds, or ulcers; punctures and abrasions of skin are apt to ulcerate; the gums become gangrenous, the teeth fall out, and the alveoli become carious. Hæmoptysis and hæmatemesis are not unfrequent occurrences.

Besides the class of symptoms now alluded to, there are others which deserve to be noticed; such as the dyspnœa, (the blood seems to be imperfectly decarbonised,) and the tendency to panting and palpitation on any active exertion.

The functions of the stomach and bowels too are almost always disturbed; the food being not properly digested or assimilated, and the alvine evacuations irregular and unhealthy. There is moreover very generally a despondency and inactivity of all the mental powers.

In the advanced stage of the disease, dysentery or some form of dropsy usually supervenes.

The rapidity with which enlargements of the spleen sometimes occur after tropical fevers, and the enormous size which they may attain, are very striking.

It is very common not only to feel, but also to *see* it extending down on a level with the naval, and even lower. In extreme cases, we are told, the diseased spleen occupies more than half the abdominal cavity, extending to the right of the umbilicus and reaching into the left iliac region.

---

*The late Dr. Twining, of Calcutta, has very justly remarked, that the constitutional disturbance which usually accompanies enlargements of the spleen, has many characters in common with chlorosis, scorbotus, and some forms of anæmia.—*Vid. Vol.* 1. *of his Clinical Illustrations of the Diseases of Bengal.*

With respect to the treatment of splenic enlargement, it is unnecessary to state that the removal of the patient from the climate, where he has acquired it, is in general by far the most efficacious remedy.

Should there be any feverishness of the system, moderate detraction of blood may be requisite, and the effects of this should be kept up by the exhibition of some active purges. When the enlargement is chronic and quite apyrexial, the use of purgatives, in union with bitters and with some form of steel, is by far the most successful treatment which can be adopted. Dr. Twining gives a formula for a compound, to which he gives the name of "spleen mixture," and which has been very successful in practice. It is thus:

℞. Pulv. jalapæ, pulv. rhei, pulv. calumbæ, pulv. zingiberis, potass. supertartratis, ää ℨj.

Ferri sulphatis, gr. x.

Tinct. sennæ, ℥iv.

Aquæ menth. sativæ, ℥x. Misce.

An ounce and a half of this may be given twice daily to an adult. Frictions over the affected side, and compression with a flannel roller, will be very generally useful at the same time.

Blisters are of very doubtful propriety. They are apt to be followed by troublesome ulcerations. The native practitioners of India have long been in the habit of employing acupuncturation in enlargements of the spleen. Dr. Twining, whom we have already quoted, remarks in reference to this remedy: "I have repeatedly punctured indolent and indurated spleen with long needles—2, 3, or 4, being introduced exactly two inches in depth—and in some instances certainly with beneficial effects."

Lastly, with respect to that panacea of some English practitioners in all visceral diseases, mercury, it deserves to be impressed on the attention of every physician, that there is not perhaps a more dangerous or destructive practice, than that of exhibiting mercurials in most splenic enlargements. The constitution of the majority of patients affected with this disease, seems to be unusually susceptible of the injurious effects of this mineral; and, therefore, as Dr. Abercrombie most justly remarks, "attempts to reduce enlargements of the spleen by mercury are generally followed by the worst consequences."—*Ed. Med. and Sur. Jour. Vol. 22.—Medico-Chirurgical Rev.*

---

8. *The efficacy of Lobelia Inflata in Inflammations and Congestions of the Mucous Lining of the Bronchial Tubes.*

To Henry J. Johnson, Esq., Lecturer on Anatomy, in the school of Kinnerton St.

Dear Sir,—I perceive from the Journals that a disease called Epidemic Catarrh, or Influenza, is very fatal in London—that it is attended with inflammation of the mucous membrane of the bronchial tubes—bears depletion badly—and that the old and infirm are the greatest sufferers. The experience and observation of a number of years devoted to the practice of medicine, have led me to two important conclusions in regard to the principal diseases of the chest.

1st. That when the serous membranes of the chest are affected, tartar emetic and the lancet are worth all other remedies put together, and those cases which will not bear the lancet, will bear tartar emetic in considerable doses.

2nd. That in those diseases affecting the mucous lining of the bronchial tubes, the lobelia inflata comes as near being a specific as tartar emetic, and the lancet in pneumonia and pleurisy. As a remedy for asthma, you are of course acquainted with the virtues of the lobelia inflata. The observation, that it holds a controlling power over inflammations of the mucous membrane of the lungs, has never, I believe, been announced to the medical world, and originated with myself. I always intended to make it known to the public. My notes of the efficacy of this remedy I left in the United States. If I had them, I could give you more definite and precise information in regard to the therapeutic properties of the lobelia in such affections. But I have thought that a bare enumeration of the principle, that lobelia inflata is almost a specific for inflammations and engorgements of the mucous membrane of the bronchi, and for spasmodic constriction of those tubes, might be attended with considerable benefit at the present crisis. I have very little doubt, that if the remedy be tested in your present epidemic, experience will establish the principle beyond cavil or dispute,—*that lobelia inflata is, for inflammations and congestion of the mucous coat of the bronchial tubes, precisely what the lancet and antimonials are for inflammations of the serous membranes of the thoraric viscera.* I have never found venesection, antimonials, or purgatives of much utility in severe catarrhal fevers. Used with moderation, the effects of such remedies are often equivocal, and in excess, always prejudicial. You have, no doubt, observed, that in violent catarrhal fevers, the mucous lining of the alimentary canal sympathizes with the inflammation of the mucous membrane of the lungs. Hurried respiration, red tongue, weak and frequent pulse occurring in catarrhal affections, especially in debilitated children and in old persons, always indicate considerable danger. This is precisely the pathological state of the system, which the lobelia inflata is so well calculated to remove. Its action is so prompt and self-evident, that there can be no mistake about its virtues. It is altogether a different medicine in this state of the system, from what it is when given to a person in health or differently affected. In the healthy state the lobelia is powerfully emetic, but in the inflamed, conjested, or spasmodic state of the bronchial tubes, it neither vomits, nor produces any sensible effect upon the great organs of secretion. It seems to spend its force in diminishing the anhelation, in lessening the frequency of the pulse, in allaying the general febrile commotion, and in restoring the balance of the circulation and excitability. Why it should do so, is as difficult to explain, as why opium is not a narcotic when it has pain or spasm to allay. I use a saturated tincture made of the leaves, stems, and inflated capsules of the plant. Dose—one or two teaspoonfuls every two or three hours, oftener, or not so often, according to the urgency of the symptoms. I mix it with about an equal quantity of the syrup of squills, or the oxymel. I have given to children, less than a year old, from 12 to 20 drops, withont producing vomiting. When the remedy vomits, it indicates that the dose is either too large, or the condition of the system is not applicable to its employment. It produces its best effects when it creates no visible constitutional disturbance. When the anhelation comes on in paroxysms, a dose or two of quinine may be safely and advantageously used in the intermission, and the lobelia in the paroxysms.

Most respectfully, your obedient servant,

SAMUEL A. CARTWRIGHT, M. D.
Of Natchez, Mississippi.

---

9. *On the Ciliary Motions in Man.* By C. T. VON SIEBOLD.—We derive this and the following articles from the British and Foreign Medical Review.

The author of this paper has continued the experiments of Purkinge and Valentin, and he has added some facts which he has himself noticed, respecting the existence of the vibrating cilia upon the mucous membranes in man. He found these cilia upon the whole surface of a nasal polypus, one hour after its removal from an adult. The length of these ciliæ was 0.0028 and 0.0022 of an English line. The motions of the cilia upon one polypus which was examined, were found to be quite regular in their rhythm: in some parts they moved backwards and forwards 300 times, in others 320 times, in others 190 times in a minute. The movements were always in the same direction; and, when once they ceased, were never resumed. By a very accurate examination, M. Siebold says, that he has ascertained, that each cilium curves its free extremity towards the part to which it moves, and that small globules of mucus, when in the vicinity of this oscillating body, are thus propelled in the same direction as the curve of the cilia. On condylomata at the entrance of the vagina no cilia could be discovered. They were also not found on the bronchial mucous membranes of those who had died of pneumonia, or of those who had suffered before death from a copious bronchial mucous secretion. In warm weather, also, it is useless to look in the human corpse for the cilia upon mucous membranes. M. Siebold has examined other membranes without finding any cilia; e. g. synovial membranes, sheaths of tendons, the internal surface of arteries and veins, that also of the vessels of the placenta, as well as serous membranes in animals, the mucous membrane of whose bronchial apparatus presented an abundance of cilia. M. Siebold recommends all those who are anxious to examine this phenomenon to first experiment upon bivalves, (Unio pictorum, Anodonta anatina, Cyclas cornea;) for in them the cilia are most evident and their motions most easily recognized, abounding as they do upon their gills, tentacula, intestinal canal, &c. Having once witnessed the structure in these animals, it will be readily ascertained in man and other animals.—*Medicinische Zeitung*, No. 28. 1836.—*Select Medical Library.*

---

10. *On the Ciliary Motions in the Brain.* By M. PURKINGE.—It has at length occurred to me to discover cilia, and their motions, in all the cerebral cavities of mammalia. In the preceding summer, while examining the Chordæ Bergmannicæ, having observed, on slices of epithelium, a stau cture similar to that of the ciliary membranes, I conjectured that it would be found to possess the same function. Accordingly I made many investigations with the view of discovering this, but in vain, until the 28th of May of the present year, 1836, when I detected the ciliary motions, in the greatest activity, in the brain of the full-grown fœtus of a sheep, about thirty hours after death. The cilia were very distinct on all the walls of the cerebral cavities, and even where they were not in motion. I traced the motions, without difficulty, through the third ventricle, into the infundibulum, then through the aqueduct of Silvius into the fourth ventricle. Here there was no motion perceptible, but the cilia themselves were distinctly visible, although somewhat shorter than in the former situations. The cilia are proportionately long pointed, (not ragged, as in the bronchi,) and vibrate thong-shaped, (peitschenformig:) we can also distinguish a layer of granules, in which they are fixed, and which can be easily stripped off without destroying the continuity of the epithelium. I recently observed them in the brain of a sheep; and Dr. Valentin saw them in the full-grown fœtus of a sow; but they could not be detected in a much younger fœtus of the same animal, probably because the parts are too fine for our gross instruments. In these observations I could remark that the cilia in the ventricles of the brain were much more sensitive and easily destroyed than in any other structure. I could not detect them in the brain of a sparrow, nor in a carp, nor in a diseased human brain. Probably they are in all parts very transient, yet readily reducible; at least, this may be considered as made out in regard to the ovaria and mucous membrane of the nose.—*Muller's Archiv.* Nos. III. and IV. 1836.—*Ibid.*

---

11. *On the Permanent Immoveable Bandage.* By Dr. Suetin.—Dr. Suetin has employed, for some time, a bandage constructed upon the same principles as those which Larrey and subsequently Deiffenbach recommend in certain cases of fracture, deformity, &c.; but he has introduced a modification which, from considerable experience, he is disposed to regard as an improvement; and he conceives that its application will be attended with benefit, in a much greater number of cases, than those in which it has hitherto been employed.

The chief advantages attending the use of the permanent immovable bandage, and which contrast it with those commonly employed, are, that when applied to a limb, it fits itself to all the inequalities of its surface, constitutes a compact covering, no one part of which can be moved without the simultaneous motion of the whole. Its action is not upon particular parts of the limb, but upon its whole circumference, and it is thus a splint in every direction. When applied, it fulfils a threefold indication: it maintains permanent coaptation; opposes constantly the action of forces which tend to disturb the relations of the reduced fracture; and effects all this, by a uniform, regular and methodic compression. These are advantages rarely to be obtained by the ordinary means, and they are such as render the bandage a valuable acquisition in many other cases, than in those of fractured limbs. And even in cases of compound fracture, where great suppuration is to be anticipated, the application of this bandage has appeared very much to limit the process of suppuration. In this observation Dr. Suetin agrees perfectly with Larrey.

Inflamed articulations, where rest is desired; diseased joints, where the object is to effect anchylosis in a favorable position, club-foot and analogous deformities of the limbs, and other obvious cases, indicate the application of this permanent bandage.

The material employed by Dr. Suetin, recommends itself by its cheapness, and with the facility with which it may at all times be obtained. The essential parts of the apparatus are a roller or many-tailed bandage, pasteboard splints, and a solution of starch in water of a thick mucilaginous consistence.

The general mode of its application, varying in some degree according to the parts to which it is applied, is as follows: Enclose the limb in the roller or bandage, and then smear its whole surface with the solution of starch. Over this again pass another roller and let an assistant spread the starch over each turn as it is made. When this has been done, apply the pasteboard splints, which must be cut and moistened so as accurately to correspond with the limb. Dr. Suetin recommends that the pasteboard should be torn instead of cut, as it has not then a sharp and irritating edge. When the posteboard is also smeared with starch, pass the bandage over the whole. Of course, in the application of this treatment to fractures of different kinds, the particular circumstances of such fractures, and the general principles of treatment will require certain modifications, which it is needless to specify. *Bulletin Med. Belge*, No. 11. Nov. 1836.—*Ibid.*

---

12. *Chronic Catarrh of the Bladder, treated by Injections.* By D. M. Devergie, Sen.—Dr. Devergie has recorded eight cases of chronic catarrh, some of long standing, which were cured by injecting balsam of copaiba into the bladder. Some of these cases had succeeded to an acute cystitis: in others the disease had gradually manifested itself and maintained throughout its chronic character. If stricture of the urethra exist, this requires to be remedied before employing injections. A moderate quantity of an emollient fluid must first be injected, to ascertain the capacity of the bladder, but not in sufficient quantity to irritate it. General means must be resorted to, to calm the inflammation and local pain, the general erethism, &c. Narcotics must next be added to the emollient injections; and these may be repeated three or four times daily. When the state of irritation of the bladder and neighboring parts is allayed, the copaiba should be injected. A dose of uniform strength is not suited to every taste. A drachm of balsam of copaiba to an ounce of barley-water is strong enough to commence with; the quantity of balsam may be increased according to its effects. The combination of narcotics with copaiba renders the latter less exciting. The balsamic injections may be allowed to remain in the bladder for a period of from ten to twenty minutes. The quantity of copaiba is to be gradually augmented; and it should not be injected more frequently than once daily, nor intermitted more than two days. The injection is to be continued until the muco-purulent secretion has quite ceased. It is necessary to guard against the occurrence of inflammation of the mucous membrane of the alimentary canal, and under such a circumstance to suspend the use of the balsamic injections.—*Gazette Medicale de Paris.*—*Ibid.*

13. *On Pessaries, and the Radical Cure of Prolapsus Vaginæ et Uteri.* By Prof. Dieffenbach.—This distinguished surgeon has long discontinued the use of pessaries in his own practice. To them he ascribes the occurrence of many diseases of the vagina and uterus, as well as of the neighboring parts; and although he admits that there may be cases in which their use is likely to be beneficial, he considers that such cases are comparatively very rare. He was led to adopt the mode of practice which he here recommends, by seeing the case of a woman, the subject of prolapsus of the vagina and uterus, in whom parts of the vagina sloughed, during its state of prolapse: the uterus and vagina were replaced whilst granulation was going on, and the result was a complete cure of the disease. The first case with which Dieffenbach met, after this, on which he was determined to imitate the natural process, was that of a woman with prolapsus of the uterus, which could be easily replaced, but as easily prolapsed, when it was not kept in by a sponge.

The operation was thus performed. The bladder and rectum were emptied; the uterus was made to prolapse, and a portion of about the size and shape of a hen's egg was removed from the left side of the vagina, the sharper end of which was directed backwards, the opposite end forwards, and came in contact with the nymphæ. The fold was then seized with a pair of forceps, the uterus being previously pressed somewhat backwards, to take off the tension of the vagina, and then dissected out with a slightly curved scalpel. The same process was repeated on the right side. The wound was cleansed, and at its hinder part two sutures were applied, the uterus was next replaced, and three other sutures were applied within the vagina. Had all the sutures been completed before the attempt was made to replace the uterus, it is possible that its reduction could not have been effected. Some little irritation followed, which ceased, however, on the removal of two of the sutures from either side. On the sixth day, all the sutures had separated.

Since the time at which Dieffenbach performed this operation, he has repeated it very often. He now employs a smaller number of sutures; usually only two, and never more than three. In many cases he uses no sutures at all, as the borders of the wound in the vagina mostly lie close in contact after the uterus had been replaced. The suture is required where there is great relaxation, and a want of irritability of the vaginal membrane; on the other hand, when the individual is robust and the vagina thick, it is better to dispense with sutures. When the surface of the vagina is mortified, it is necessary to fill it with charpie. Tepid mucilaginous injections should be used for some days, and after these, cold water. If, when cicatrixation is going on, there is no evident narrowing of the vagina, a compress of charpie smeared with a resinous ointment, and the repeated application of the lapis infernalis, should be employed.

Dieffenbach has often removed the fold from the vagina after having replaced the uterus, by drawing a portion of the former outwards, and cutting it off by a knife, with a sawing motion. This is a far easier mode of operating, but great care is necessary not to injure the bladder or rectum, which may happen if the fold of the vagina, when tightly stretched by the forceps, should be cut off too near its base. Sutures are not employed in this case.

The position of the patient in the operation above described, should be the same as that for lithotomy. The state and relations of the rectum and bladder with the vagina and uterus should be ascertained, previous to the operation; of the former, by means of the finger, of the latter, by Desault's silver catheter. The catheter sometimes draws off a quantity of retained urine; the evacuation of the bladder being often rendered very difficult by the prolapse of the uterus.—*Medicinische Zeitung*, No. 31. 1836.—*Ibid.*

—

14. *On the use of the Plug in cases of Placenta Prævia.* By Dr. Albert, of Wiesentheid.

*Case.*—A robust, plethoric woman, mother of seven children, was attacked with slight hemorrhages from the vagina, which recurred every three or four days, from the latter end of the eighth to the middle of the tenth month of her pregnancy, when the discharge became more profuse. The placenta was found centrally situated upon the os uteri, which was dilated to about the size of a shilling. Although she was not exhausted, yet, as her condition was not free from danger, Dr. A. prepared a plug of fine linen, which was oiled, and passed up the vagina against the os uteri; the vagina was also stuffed with charpie, and the whole secured by a proper bandage. The patient was desired to remain quiet in the supine posture, with her knees together, and she was left for some hours under the charge of a midwife, with proper instructions. After a considerable period, Dr. A. was again called; expulsive pains having come on, during which the plug produced much straining and tension. On removing it, the liquor amnii escaped, and a small quantity of blood in clots. The right edge of the placenta was detached, and hung down against the left side of the vagina. The os uteri was fully dilated, and the largest circumference of the head had passed through it. The child was expelled in about half an hour; the placenta followed immediately, and nothing occurred to disturb her getting up. The child was alive and active.

[We do not approve, says the British and Foreign Medical Review, from whose pages the above article is obtained, of the plug used by Dr. A., although sanctioned by Mr. Burns's authority. It is far inferior to the sponge plug, as recommended by Dr. Dewees. This is introduced and removed with far greater ease, and, being much softer, is retained in the vagina without inconvenience by the patient. Our objections to the linen plugs are illustrated in Dr. A's next case, where it is required to be altered twice before it could be retained by the patient.]—*Neu Zeitschrift fur Geburtskunde*, Vol. iv., Nos. 1 and 2. 1836.

—

15. *Two cases of complete recovery from laceration of the posterior part of the Vagina and Cervex Uteri.* By J. Toogood, Esq., Surgeon to the Bridgewater Infirmary.—These cases are interesting, from the rareness of recovery after such an accident. They are related with commendable brevity. In the first case there was "very extensive laceration of the posterior part of the vagina, which extended to the cervex uteri:" both Mr. T. and another surgeon, believing the case hopeless, "passed the hand freely through a large rent into the cavity of the abdomen, and felt the different viscera distinctly, and the abdominal aorta." Although given up, this woman gradually and rapidly recovered. Mr. T. is disposed to attribute the recovery to the great attention paid to the case, and to the *omission* of blood-letting on the accession of reaction with abdominal pain, &c. He was so satisfied that the loss of even a small quantity of blood, although attended perhaps with immediate relief, would in all probability have been fatal, that he "determined to trust to opium, cordials, nourishment, and perfect quiet;" and the result seemed to justify the treatment.

In the second case, there was also "a considerable laceration of the posterior part of the vagina, in which the cervix uteri was involved, and the intestines were lying in the vagina." The treatment is not given in detail, but it would appear that large opiates were the early remedies depended on. This patient also completely recovered.—*British Annals of Medicine*, Jan. 20, 1837.—*Ibid.*

---

16. *Section of the Tendo Achillis.**—Dr. Cunier, one of the collaborators of the Bulletin Medical Belge, a distinguished physician to the Belgian army, having obtained permission from the government to travel into the south of France for the restoration of his health, profited by his stay at Montpellier to learn the doctrines of the faculty attached to its celebrated school. The following is from one of his communications to the journal in question.

This operation was performed for the first time in 1784, under the inspection of Thilenius,† a physician in the environs of Frankfort; it was subsequently performed by Sartorius,‡ and Michaelis.§ After them it fell into oblivion, until Delpech cured by the section of the tendo Achillis a club-foot *(pied equin)* which had resisted all the means made use of to restore it to its proper position. This eminent surgeon remarked, that in most cases of rupture of this tendon, it was impossible to bring the two extremities of the divided tendon into exact contact, but that the tendon did not take place less effectually by the interposition of a fibrous substance which becomes organised, and is sufficiently solid to prevent the exercise of the limb from suffering except from the elongation of the part; thence he deduced that what happened in cases of rupture would also take place in cases of section, and that the interposed fibrous substance would be susceptible of yielding to the efforts of extension—*(Chirurgie Clinique de Montpellier*, vol. i.). Performed according to these ideas, the section of the tendo Achillis was followed by the most satisfactory success; the young man on whom the operation was performed was known to all Montpellier; not only is

*Bulletin Medical Belge, Mai, 1837, p. 60.
†Thilenius Medicinische und Chirurgisch. Bemerkungen, Frankfort, 1789, p. 335.
‡Sartorius, in Siebold's Sammlung, vol. iii., p. 258.
§Michaelis, in Hufeland's Journal, vol. vi., 1811.

the deformity corrected, but he walks very well. Yet the sarcasms with which Delpeck was loaded caused him to abandon this operation. But his surgical notion was turned to account by some German surgeons who resumed it with energy: Chelius *(Traite de Chirurgie)* Radius *(Dict.)* *Zimmermann (der Klump-fuss*, etc.) recommend the section of the tendo Achillis, and sometimes also that of the tibialis anticus; in the posterior club-foot far advanced, and in adults, dividing the tendon, according to them, is the only means of remedying the deformity. Performed lastly by Stromeyer, Stoltz, and several others, the operation has performed a complete cure. The following is the mode employed by Stromeyer:—The patient being seated on a table, and presenting towards the surgeon the side on which the operation is to be performed; one assistant holds the knee, another bends the foot in such a manner as to have the tendon at full stretch, so as to facilitate the section. A very narrow pointed bistoury, curved so that the edge presents a convexity, is introduced flat three inches above the insertion of the tendon, between this and the tibia. The back part of the bistoury being then turned towards the bone, and the edge of the tennon, he cuts, with small saw-like movements, the tendon, which soon divides. He does not re-unite the extremities, but proceeds to restore the foot to its natural position.

*Delpech's mode.*—When this surgeon performed the operation, the patient was laid upon his belly, and the bistoury was introduced flat behind the tendo Achillis, so as to produce on each side a wound of an inch in breadth. The instrument having been withdrawn, he introduced into the wound a convex knife, whose edge was turned towards the tendon, which was divided without implicating the skin situated above it. The operation being ended, the two extremities of the tendon were approximated, and kept in contact by an appropriate apparatus. To this end two straps were strongly applied to the knee above and below the patella: to these straps two splints were fixed, as for fractures, which extended down to the level of the sole of the foot: to this was fixed the sole of a shoe, which, by means of some turns of a bandage, kept the foot in a proper position.

Suppuration, exfoliations, &c., which Delpech observed, led him to pronounce subsequently (*Orthomophie*, vol. ii., p. 330,) that a tendon to be cut ought not to be denuded as in the two large openings which he had made; that the section ought to be made in an oblique direction, and not by a parallel section of the skin: according to Chelius *(Trad. de Pigne*, vol. i., p. 480,) the skin should be opened on each side of the tendon. M. Bouvier has lately (12th Sept. 1836) proposed a new method to the Academy, consisting in the introduction, under the skin covering the tendon, of a kind of needle sharp on one side, by means of which it may be divided, either from without inwards, or from within outwards. This was fulfilling simply the indications of Delpech, whose mode, says M. Bouvier, (*Seance de l'Academie*, last 12th of Sept.) leaves something yet to be desired. This may be more correct as to the mode employed by Delpech, but not with respect to that which he recommends in his *Orthomorphie*.

M. Serre has just performed successfully the section of the tendo

Achillis in a case of club-foot. The state of the patient at his entrance into the Hospital of Montpellier is described in a thesis learnedly reasoned on, and ably defended, by M. P. Chollot, March 18th, 1837; I will quote the words of the author:—"There is at this period, in the wards of the Hotel-Dieu Saint Eloi, an individual who presents a well-marked club-foot. It is nearly three years since he received a cut with a sickle across the calf of the leg; the wound was long in cicatrising, and when this was accomplished, the patient experienced acute pains in the wounded region; then he observed the parts to increase in size; the pains were of a nature to indicate the formation of pus. In fact, after the expiration of some time, an abscess was obliged to be opened for him, and a considerable quantity of pus discharged from it; the wound was not long in being completely cicatrised. What was his surprise when, wishing to use his limb, he found this quite impossible! During the whole course of his disease he had kept, in order to avoid pain, his leg bent on his thigh, and the foot had not been kept extended. At the present time, the phalangian extremity of the metatarsal bones in the point of support in walking."—*P. Chollot, de Fontoy (Moselle), Du Pied-bot Posterieur, These*, No. 27.

M. Serre undertook the operation in the following manner. The patient being placed upon his belly, the tendon was laid hold of by two fingers of the left hand. A very narrow bistoury was introduced flat, in front of the tendon, as far as the skin of the opposite side, which he was cautious in not injuring. A narrower bistoury than the first, and probe-pointed, was then carried along the blade which had been first introduced; the latter was withdrawn after the probe-pointed bistoury had been turned so that the back was presented towards the bone and the edge towards the tendon; little saw-like motions were made by the instrument, and the complete section was soon apparent from the noise which it produced. A piece of pasteboard was placed upon the instep and kept there by means of some turns of a bandage, in order that the foot might rest in such a position as to keep the extremities of the tendon at the distance of about half an inch from each other. The wound resulting from the introduction of the instruments bled but little; united by means of adhesive plaster, it closed the next day. The eighth day after the operation, the organised fibrous substance was submitted to an extension carefully managed and increased gradually. The fortieth day has arrived; the foot is restored to its natural position; the intermediate organization is perfectly solidified, to the extent of two inches and a half, by which the limb was previously shortened. The operation is crowned by the most complete success. There has not been the slightest inflammation.

Professor Serre proposed at first to penetrate between the tendon and the skin, and thus to perform the section *from without inwards;* but an anatomical inspection of the part induced him to abandon his project. There is indeed a circumstance on which M. Bouvier had not thought, in advancing that the incision may be practiced in this manner; the external saphena vein and the nerve of the same name might easily be divided, and thus the endeavor to restore the foot to its natural position might be baffled. In performing the incision, M. Serre did not in any wise expose the tendon to the air; he has been satisfied with this plan according to the recommendation of Delpech; the latter re-united the extremities; M. Stromeyer left them too far separated; in the present case the middle course has been adopted.

*American Medical Intelligencer.*

---

17. *Medical Education in Berlin.*—In the "*Index Lectionum,*"—or index of the lectures for which we are indebted to a valued correspondent in England—we have a list of the different lectures* delivered in the University of Berlin, by the three sets of teachers, the *professores ordinarii*, the *professores extraordinarii*, and the *privatem docentes* or "private teachers," in the different faculties. This, to one unacquainted with the extent to which the business of teaching is divided and subdivided in the German universities, is absolutely astounding. The medical part of this index alone concerns us: from it we extract the manner in which the student may occupy his hours during the collegiate year. Four years must be spent by every candidate for a degree, and it is thought that in future five may be required.

LECTURES.

7 to 8 A. M.—Reich (P. E.†), special pathology and therapeia. Ascherson (P. D.) general and special surgery.

8 to 9.—Horn (P. O.) on the diagnosis and cure of syphilitic diseases; and on the special therapeia of acute and chronic diseases. Link (P. O.) on pharmacology. Schult (P. O.), on physiology. Wagner (P. O.), special pathology and therapia. Eck (P. E.), general and special physiology. Froriep (P. E.) surgical operations, aciurgia, and surgical anatomy. Kluge (P. E.) clinics of syphilitic diseases. Reich (P. E.) special pathology and therapia. Wolff (P. E.) clinical exercises. Isensee (P. D.), special pathology and therapia, alternately.

9 to 10.—Hecker (P. O.) special pathology and therapeia. Iüngken (P. O.), clinical ophthalmic exercises. Kluge (P. E.) clinics of syphilitic diseases. Osann (P. O.), on the aids to be afforded where life is suddenly endangered. Schlemm (P. O.), on splanchnology. Dieffenbach (P. E.), universal and special surgery. Froriep (P. E.), aciurgia and surgical anatomy, Kranichfeld (P. E.) medical methodology. Troschel (P. D.) general and special surgery.

10 to 11.—F. Hufeland (P. O.), semeiotics. Kluge (P. E.) general surgery; also, theoretical and practical part of obstetrics, and doctrine of fractures and luxations.

10 to 11½.—Rust (P. O.) clinical exercises.

11 to 12.—Bartels (P. O.) clinical medical exercises. Kluge (P. E.) elements of the art of obstetrics; also, general surgery; also, doctrine of fractures and luxations; also, the theoretical and practical parts of obstetrics.

12 to 1.—Bartels (P. O.), clinical medical exercises. Link (P, O.),

---

*Index Lectionum quæ auspiciis Regis augustissimi Friderici Guilelmi tertii in universitate litteraria Friderico Guilelma par semestre Hibernum a MDCCCXXXV. MDCCCXXXVI, a die xix. Octobris instituentur. Berolini. 4to, pp. 30.

†P. E. affixed to a name means Professor Extraordinarius; P. O., Professor Ordinarius; and P. D., Privatim Docens.

on cryptogamous plants. Rust (P. O.), general surgery. Schlemm (P. O.), articular ligaments and aponeuroses; also, osteology. Schultz (P. O.), officinal plants; also, materia medica, with the doctrine of medical formulæ. Casper (P. E.), forensic medicine. Barez (P. D.) clinical exercises on the diseases of children. Phœbus (P. D.), general anatomy and histology. Romberg (P. D.), diagnosis, with demonstrations on the sick; also, doctrine of nervous diseases. Troschel (P. D.), helcology; also, doctrine of diseases of the teeth.

12 to 1½.—Ehrenberg (P. E.), comparative physiology.

1 to 2, P. M.—Hecker (P. O.) medical encyclopædia and methodology. Horkel (P. O.) general physiology. C. Hufeland (P. O.), clinical exercises. F. Hufeland (P. O.) special therapeia. Osann (P. O.) polyclinics. Eck (P. E.) contagious diseases. Reich (P. E.) on the diseases of evolution. Trüstedt (P. E.), clinical medico-chirurgical exercises. Phœbus (P. D.), physical anthropology.

2 to 3.—C. F. Gräfe (P. O.), surgical and ophthalmic clinics. Müller (P. O.), anatomy of the human body in health. Casper (P. E.), first part of special pathology and therapia. Ideler (P. D.), diagnosis and cure of mental diseases.

3 to 4.—C. F. Gräfe (P. O.), universal aciurgia. Muller (P. O.), anatomy of the organs of sense. Wagner (P. O.), political medicine; also, forensic medicine. Casper (P. E.), first part of special pathology and therapeia; also, the other part. Isensee (P. D.), special pathology and therapeia.

3 to 4½.—Kranichfeld (P. E.), special therapeia of the eye.

4 to 5.—Busch (P. O.), obstetrical clinics. Bartels (P. O.), aphorisms of Hippocrates; also, pathology and therapeia. Hecker (P. O.), history of medicine. F. Hufeland (P. O.) general pathology. Iüngken (P.O.), diagnosis and cure of the diseases of the ear and eye. Wagner (P. O.), practical exercises on the institution of forensic medicine. E. A. Gräfe (P. D.) general and special surgery. Isensee (P. D.), on certain new remedies and methods of cure. Mitscherlich (P. D.), materia medica. Oppert (P. D.), doctrine of the diagnosis and cure of syphilitic diseases; also, general therapeia. Wilde (P. D.), obstetrics. Isensee (P. D.), special pathology and therapeia.

5 to 6.—Busch (P. O.), obstetrics; also, elements of the art of obstetrics. Bartels (P. O.), pathology and therapeia. Iüngken (P. O.), diagnosis and cure of diseases of the ear. Osann (P. O.), materia medica. Kranichfeld (P. E.), Hygiène. Ascherson (P. D.), art of bandaging. Dann (P. D.), special pathology and therapeia. Nicolai (P. D.) medical police, and the medical regulations of Prussia; also, general pathology, symptomatology, and 'exploration' of the sick.

6 to 7.—Busch (P. O.), obstetrics. Angelstein (P. D.), doctrine of operations on the eye; also, universal and special ophthalmiatrice. Isensee (P. D.), medical and forensic psychology.

7 to 8.—Dieffenbach (P. E.), surgical operations. Eck (P. E.), physiological part of the institutes of medicine. Angelstein (P. D.), practical course of operations on the eye. Dann (P. D.), diseases of the ear. Ideler (P. D.), clinics of diseases of the mind.

Some of the professors deliver also private courses; and, where they appear to occupy two subjects in the same hour, it is not on the same days of the week. Some, too, of the lectures, it will be observed, occupy two hours.

Each candidate for graduation, besides being required to pass rigid examinations on the different branches of the healing art, held in the Latin language, is compelled to compose and defend a Latin dissertation on some acceptable subject. These are generally of a superior order, in consequence of the thorough medical education, which four or five years of study is capable of effecting. To some Berlin dissertations before us, a short "vita," or biography, of the candidate is appended. We cite one from a dissertation—"De Dilatatione Ventriculi," defended in December, 1835,—as a specimen. It is a kind of history of the education of the author.

I, Walter John Fackeldey, born at Embrica, on the 8th of September, 1809,—my father's name F. Fackeldey, and my mother's Joanna, of the family of Van den Brock,—profess the catholic faith. My first rudiments of letters were acquired in my native town, under an evangelical pastor, Zur Nieden, a much prized preceptor; thence I went to the gymnasium at Cleves, which was under the directorship of the excellent Nagel, where I studied for four years. I then went to Munster, where my testimonial of fitness having been received by the director of the medico-chirurgical school—the estimable Bodde—I attended the following lectures:—*experimental physics*, by the celebrated Roling; *chemistry*, by the estimable Bodde; *anatomy and dissections*, by the distinguished Tourtual; *general pathology*, by the celebrated Busch; *zoology*, by the distinguished Beck; and *physiology*, by the celebrated Heindorf. At the expiration of the half year I entered the University of Giessen, and attended the following lectures:—*botany*, by the celebrated Wilbrand; *psychology and logic*, by the distinguished Koch. I then went to Bonn, and having taken out my inscriptions with the dean, I attended the following courses;—*general pathology, methodology; and encyclopædia*, by the celebrated Müller; *general therapeia*, by the distinguished Harless; *anatomy and dissections*, by the celebrated Meyer and Weber. In the year 1832, I went to Halle, and was admitted by the honorable Prorector Hefter, and the excellent Meckel, Dean of the Medical Faculty. My preceptors were the following distinguished individuals:—Germar, on *mineralogy and crystalography;* Friedlander, on *materia medica and the art of prescribing;* Gerlach, *psychology;* Krukenberg, *pothology and special therapeia;* Blasius, *luxations and fractures;* and Niemeyer, *theory of obstetrics.* I followed the clinical school of these illustrious men,—Krukenberg being physician, and Blasius surgeon-occulist. In the autumn of the year 1834, I went to Berlin, and was admitted into the university by the honorable Rector Steffens, and the distinguished Busch, Dean of the medical faculty. There I attended the following clinical lectures: the *obstetrical* of the distinguished Busch; the *therapeutical* of the distinguished Bartels and the celebrated Wolff; and the *opthalmiatric* of the distinguished Iüngken.

To all these individuals I shall ever owe affection and gratitude.

My desire is, that after I shall have passed my examinations *(tenta-*

*minibus)*, philosophical, medical, and rigorous, before the honored medical faculty, and have publicly defended this dissertation, and my theses, to have the *summi honores*, in medicine and surgery, conferred on me.—p. 32.

The philosophical examination, to which allusion is made in the last sentence, is on chemistry, physics, zoology, botany, mineralogy, and philosophy, by a board consisting of members of the Faculty of Philosophy of the university. The *tentamen medicum* is an examination, conducted, both in writing and *viva voce*, before the dean; and the *examen rigorosum* takes place before the medical faculty, and is on all branches of medicine.

The "Theses," which the candidate has to defend publicly, and of which mention is made in the "*Vita*" of Dr. Fackeldey, embrace various topics. In his case they were as follows:—

1. The torsion of arteries should be abandoned.
2. Many diseases of the chest cannot be known without ausculation.
3. The asthma of Millar and the tussis convulsiva differ greatly from each other.
4. There are no specific remedies.
5. Hectic fever does not forbid amputation.

&c. &c.

---

17. *The Medical Profession.*—There is much truth in the following pertinent observations from the pen—if we mistake not—of a distinguished lawyer, David Hoffman, Esq., of Baltimore. The whole work, in which they appear,* is well worthy of attentive perusal. It is evidently the production of one accustomed to observe accurately, and to reflect deeply.

"THE MEDICAL PROFESSION.

"We could easily say many very civil and truthful things of the doctors; but this would be, what the lawyers call, a '*departure*,' for it must have been perceived by our readers, that we have not been in pursuit of the virtues, but mainly of the follies, incommodities, vulgarities, and vices of men, manners, and things! What boots it, moreover, to tell a man, or class of men, of their amenities, their clevernesses, their virtues; have they not already sufficient vanity, and are they not apt enough to set their amiable qualities and goodly possessions in the highest possible relief? *Credo di si.* What, then, can now be said in brief words, and, of course, with strict adherence to truth, *against* many (not all) of the doctors? We opine, that they are sometimes too apt to make their calling a plea for an almost perpetual absense from church! They seldom say a kind word for each other! They are, of all critics, the most acrimonious in their reviews of each other's productions! They are instinct with the most inconvenient and refined etiquette, as to what they call professional deportment in respect to each other's patients! Their regard for rigid veracity sometimes suf-

*Miscellaneous Thoughts on Men, Manners, and Things. By Anthony Grumbler, of Grumbleton Hall, Esq., (with a motto.) 12mo, pp. 374. Baltimore, 1837.

fers insensibly, from an over-nice and ill-judged, but still, well-meaning assentation to the wishes of their patients, and in tenderness to the feelings of their relatives! They too often neglect small complaints, when they attain to a high and prosperous practice! They are too fussy and pretending, when not in full practice! When very popular they are sometimes very presumptuous! They are generally too grandiloquent, and too garrulous in the sick-room, loving the gossip of the town, and forgetting, as Menandes of old justly observed,

'A prating doctor is a new disease unto the sick.'

They read too little, after they have attained a goodly share of practice! They generally charge too little for their services, and sometimes *underbid* their professional brethren! They often pay too little attention to *dietetics*, and are apt to estimate too lightly the *veletudinary* state! They are too much given to systems and theories, failing to consider their science, as it certainly is, of all others, the most strictly *inductive!* They are not sufficiently posted up in the modern and current history of their science; or, what my Lord Bacon calls the *narrationes medicinales*, and to the neglect of which, in his time, he attributed the slow progress of physic! They practise too much upon the maxim of Bolingbroke, that whilst *plain truth* may influence half a score of men, *mystery* will lead millions by the nose! They seldom know how to cast off, even for an occasion, their professional identity—their 'exoteric trappings'—and cannot lay them aside, as the Lord Treasurer Burleigh used to cast off his cares of state, when he put off his outer garments, saying, as he laid down his gown, "Lie there, Lord Treasurer.' In fine, as a genus, as an order, as a species, as a variety, however strongly marked they may be with many very clever qualities of head and heart, they still are doctors!

"It hath been said, with perhaps more wit than justice, that all orders of men hate whatever tends to diminish the characteristic features of their profession. Hence it is supposed that lawyers adhere to the letter of the law, and hate equity; that divines undervalue morality, because so liable to be substituted for religion; that apothecaries hate quacks, because their medicines are panaceas; that physicians hate regimen when opposed to medicine; that the physician, surgeon and apothecary agree well enough when kept asunder, but that when they attempt (as did Master George Thompson, after the great plague of 1665) 'to make *unity* of that which was then a *trinity*,' they were all hissing at each other like so many serpents, and have continued so ever since, wherever the professions are thus united, or attempted to be; that the empirical practitioners hate the book-men, and they, in turn, detest the empirics, and so on. Certes it is, that there hath been, here and elsewhere, but little harmony among the sons of Esculapius, however denominated: and as little uniformity in practice, as well as in theory, as in any other science, art, or profession whatever. A certain Dr. Stevenson was for selecting, out of the whole materia medica, but eight officinals, and consigning all the rest to be poured into the streets! Dr. Morton, who introduced the use of bark, had to encounter a severe ordeal before he established its use. 'By this *short method*,' says he,

'of curing fevers, physicians lose opportunities of picking the pockets of their patients.' Lady Mary, when she introduced inoculation, encountered no little opposition. Jenner, also, when he struck out one of the foulest of diseases from the catalogue of human woes, was not without his enemies. James's powders, now near a century old, stemmed such a torrent of prejudice as had well nigh destroyed them and their inventor. Nay, if the history of medicine be looked into, it will be found that scarce any theory, any remedy, any prophylactic given to the world, was ever, in the first instance, fairly, and dispassionately examined by the profession; and this is so remarkably the case as to have induced Mr. Hobbs to say of Dr. Harvey, who discovered the circulation of the blood, that he was the 'only one that conquered *envy* in his life time, and saw his new doctrine established'—Herveius solus, quod sciam, doctrinam novam, superatâ invidiâ, vivens stabilivit. The quarrels, the abusive pamphlets, the severe criticisms, and hard speeches, that have marked the course of American physicians, during even our short and brilliant medical career, would fill many volumes; and whilst they are very creditable to the head, are far from being so to the heart. They sully the reputation and just weight of a highly useful and learned profession; and inculcate very prejudicial doubts in the minds of young physicians, at the very commencement of their studious career."

---

18. *Statistics of Hospitals.*—The following facts relating to medical statistics are taken from the British Medical Almanack.

1. *Do Examinations after Death diminish the number of Applicants for Admission into Hospitals?* Most certainly they do not. The alarmists would fain terrify us with prognostications of loss of hospital patients, from the prevalence of dissections, or from the operations of the Anatomy Act. Benevolent and timid people are very apt to overrate the sensibilities of the poorer classes. No doubt their ignorance makes them liable to prejudice. But even prejudice yields to the operation of necessity, and the sense of interest. The poor may at first raise a clamor against dissection: but when their lives are at stake, when present comfort and medical attendance, with the possibility of future dissection, are balanced against distress and no medical relief, the decision is pretty surely in favor of the former. A small knowledge of human nature would lead us to anticipate this result. Experience, both on the Continent and in this country, has proved it. Indeed, the framers of our Anatomy Act might safely have taken a bolder step than they did, and the working of the bill would probably have been better than it actually is. Political considerations interfered, and still interfere, with the insertion of compulsory clauses in that Act. We say that experience has decided, that the knowledge on the part of the poor, that examinations after death are the rule of a hospital, does not diminish the number of applicants for admission into that institution. Perhaps there is no hospital, in London or out of it, where examinations after death are more constantly and more systematically pursued than they are in St. George's Hospital, Yet the number of applicants far exceeds the capability of accommodation for them, although that hospital contains upwards of three hundred beds. In other hospitals the case is just the same.

"At a hospital in an agricultural district, the house surgeon for the time was allowed to examine every body—in six months eighteen or twenty bodies were carefully inspected, without asking permission from the friends; this had not been the custom before, yet the average number of in-patients increased that year to 100, the admissions to 1112; the average number of in-patients the year before had been 76, the total admissions 837. The connexion of a theatre for dissection with hospitals necessary excited more prejudice formerly, than the simple inspection of the body excites now; yet experience proved that this had no influence whatever in diminishing the number of applications for admission. Cheselden commenced courses of anatomy at St. Thomas's in 1711; in 1706 it contained 362 patients, in 1717 the number had increased to 420. For other reasons it is not desirable that theatres for dissection should be immediately connected with hospitals; but this circumstance has not at all been found to diminish the number of patients at St. Thomas's, Guy's, St. Bartholomew's, the London, or any other institution. The number of applicants for relief at a hospital depends on the *kind manner* and *reputed skill* of the officers; the poor have sagacity enough to discover that the person most zealous in investigating their diseases is best qualified to relieve their sufferings."

We may set it down as a certain fact, that neither systematic examinations after death, nor the connexion of a school of anatomy with any hospital, will in any degree curtail the amount of its patients.

A. *Numbers of Patients and of Deaths in the Hospitals of Great Britain.*—"Besides 182,847 seen at the dispensaries and as out-patients, more than 24,000 patients are treated, and 2,100 die within the walls of the London hospitals. According to the last printed reports, 30,000 are admitted, and 1780 die in the country hospitals; in the Scotch infirmaries about 6,900 are treated, and 670 die; in Ireland, it appears from official returns and Mr. Phelan's work, that 8126 are treated in the country infirmaries, 6855 in the hospitals of Dublin, and nearly 15,000 fever patients in the establishments destined for their reception. The cases of disease placed every year, in the public institutions of the country, under the observation of the medical officers, amount, therefore, to at least 90,000, 6,300 of which terminate fatally, and may be submitted to necroscopical investigation. At a moderate estimation, the dispensary and out-patients are four times as numerous. The sums expended in supporting the medical charities of the country are enormous: the value of the labor devoted in them to the treatment of the classes unable or able to pay for medical advice, can scarcely be calculated."

B. *Advantages of due Statistical Returns from Hospitals.*—It is unnecessary to dwell on the advantages that would accrue from accurate and well-digested statistical hospital reports. It is only by the combination of particular facts and of general results that much good can be done to medicine. The time has arrived when a general and a well-arranged system of hospital reporting must begin to attract serious attention. There are some suggestions in the work before us, which we will lay before our readers, and particularly before the medical officers of hospitals.

c. Dr. Clifton, who published a work intitled the State of Physic, in 1732, proposed the following plan:

"In order, therefore, to procure this valuable collection, I humbly propose, first of all, that three or four persons should be employed in the hospitals (and that without anyways interfering with the gentlemen now concerned) to set down the cases of the patients there from day to day, *candidly* and *judiciously*, without any regard to private opinions or public systems, and at the year's end publish these facts just as they are, leaving every one to make the best use he can for himself. Would not some such method as this let us more into the nature of diseases in a few years than all the books of theories, or even the books of observations, hitherto published? Certainly it would: and yet if proper encouragement were given, it is not at all unlikely, but that persons enow would soon be found, every way qualified for such an undertaking. And even if good salaries were allowed them, and every thing made as easy and agreeable to them as they could desire, the benefit the public would receive from them would vastly more than balance the expense."

There is an objection to the publication of reports from a hospital in a separate form. Such a form of publication is attended with much trouble and some expense, and, after all, as it costs money, will not be very widely circulated. The best mode of rendering the facts that occur in hospitals generally available and useful to the profession, would be their regular and periodical publication in the Journals. We would suggest the combination of reports at short and at long intervals—in a weekly and in a quarterly journal. We should have no objection to open our pages to a well-conducted system of hospital-reporting. Sooner or later, some plan of the sort must be adopted.

The object, however, at the present moment is to agree on what is the best plan of *statistical* reporting. This is not so obvious as at first sight it might appear, and the Editor of the Almanack makes some observations, which are well worthy of attention.

"The first step in medical statistics, after having determined the mortality, is to ascertain the number of attacks of sickness, at different ages, to which a population is liable, and the numbers constantly ill. Hospitals throw no direct light on these questions, or on the absolute mortality and duration of cases. But hospital statistics may show the mortality and expected duration of different diseases at several stages of their progress, and the extent to which their mortality and duration are diminished by remedial means. To make the series of observations perfect, the cases should be traced from their origin to the termination; but patients only enter the hospital at a certain period of their complaint, on the 10th, the 20th, or the 30th day, &c.; and before this time, out of 1000 attacked, 500 perhaps have either recovered or died: you then can only have to deal at the hospitals with the remaining 500, whose history you may retrace, and then follow for a mean term of thirty, forty, or sixty days; when a certain number still remain ill, but the majority are discharged cured, relieved, or dead. Because hospital cases only come under notice after a certain time has elapsed, and are not all followed to the end, it has been thought they afford little instruction. We have reason to entertain a different opinion: at the same time, let it be constantly borne in mind that the mortality is merely the mortality for certain periods of disease: from the twentieth to the fifty-sixth day, for instance—it is not the entire mortality of the class of cases. To represent this, all the deaths that occur after leaving the institutions, should be added; thus augmenting the mortality, which is however lowered, on the other hand, by another powerful cause. Admit that 1000 laborers are attacked by disease; all repair to the infirmaries, who remain ill, on an average, twenty days; but before that time, 280 have recovered, and twenty died: in the institutions 700 remain under treatment till the fifteenth day, and during this period fifty die, and 450 recover; 200 leave uncured, and of these, ten die. The mortality of the cases would be 8 per cent.; the mortality of the cases, while in the institution, was fifty in 700, or 7 per cent. It may happen that the mortality in the two instances coincides by a sort of mutual compensation between the excess of previous recoveries and the subsequent deaths. Nearly the same reason applies to the duration of cases."

It cannot be expected that the deaths which occur after dismissal from the institutions can be reported on. The day of the disease on admission—the occurrences whilst in hospital—and the day of discharge or of death, will probably constitute the main statistical facts that we can obtain.

D. It would be advantageous if the annual reports from all hospitals referred to the same period—from the 1st of January to the 31st of December. As it is, the reports of some hospitals start from Lady-day, or Midsummer, and thus accurate comparison with other hospitals is interfered with.

2. "*The numbers treated yearly is the mean of the numbers admitted and discharged.* The total number of in-patients in the year is generally published in this manner:

In-patients admitted from Jan. 1st, 1833, to Jan. 1st, 1834.

| | |
|---|---|
| Remaining, Jan. 1st, 1833, - - | 46 |
| Admitted since, - - - - - - | 342 |
| Total, - - - | 388 |
| Discharged cured, - - - - - | 200 |
| Relieved, &c. &c. &c. - - - | 142 |
| Remaining Jan. 1st, 1834, - - | 46 |
| Total, - - - | 388 |

In some reports it seems to be implied that 388 patients have actually been treated in the year. This is an error: you have already counted the forty six *once* in the Report of 1832; and of the 342 admitted, you will in the same way count forty-six a *second* time in the year 1834. The forty-six patients in the house at the beginning of the year have, on an average, remained half their time—for instance, twenty days; they will probably still remain twenty days; they may, therefore, be regarded as half-treated, the same as half the number fully treated; add, then, 23 to 342, and you obtain 365; but forty-six remains at the end of the year only half-treated; and you have already counted them for fully treated among the 342, as a correction, therefore, deduct 23 from 365, and the original number 342 remains. 342 patients have been admitted, and 342 have been discharged in the year; this is, therefore, the number annually treated. The number treated may be deduced from the number admitted and remaining; or from the number discharged and remaining: and generally, when several years are taken together, and the size of the hospital remains the same, the number discharged or admitted, exclusive of the number remaining, represents the number treated, with sufficient accuracy."

3. *Exaggerations of Numbers of Patients.*—Hospital annalists are as anxious to multiply the number of their patients, as doctors are in private practice. Thus at some hospitals a patient is re-counted whenever he changes his condition from that of out-patient to that of in-patient, or the contrary—or he is obliged to take out a fresh ticket every two months, and is reckoned as a fresh patient, "an ingenious zootomical device," and well calculated to knock statistics on the head. All this is, in a scientific point of view, reprehensible.

4. Patients are reported as "cured, relieved, uncured, or dead." The term cured should be employed in a uniform and rigorous sense. It is suggested that another head should be generally added—"discharged for non-attendance," a discharge which should take place, if nothing is heard of the patient for a fortnight. These non-attending patients are very convenient to reporters, and serve for cured in a wholesale way.

5. "*The annual mean number of in-patients under treatment may be determined by counting the patients every day in the year at the same hour—for instance,* 12 *to* 1 *o'clock—and dividing the sum by* 365*:* the average number of dietaries, or of persons *sleeping* in the house, would furnish the same results. When patients are admitted and discharged on a particular day, the patients discharged should be counted, if the admission and discharge take place after dinner; the patients admitted, if before; both should never be reckoned. Approximation to the average number treated may be obtained, by adding together the number in the house on a fixed day of every week or month, and dividing the sum by fifty-two or twelve. The average number of patients under treatment is exaggerated by counting the *beds* actual or possible; by selecting the greatest number ever in the house; or by taking one day in the week, and counting, in addition to the patients then resident, all who *have been* in the house during the six preceding days. When the absentees are duly erased from the books, the average number of out-patients can be calculated in the same manner as the in-patients; the mean number that *attend* should also be ascertained. *None of the hospitals in the metropolis*, except the *London Hospital*, publish the average number of patients under treatment; but this essential statistical element is published by the Cambridge, Winchester, York, Gloucester, Exeter, Nottingham, Salisbury, Salop, Chester, Canterbury, Bedford, and Colchester Infirmaries. It is the main feature of a report, and alone controls the expenditure. It is either suppressed from imperfect knowledge or inattention; to allow the extent of the institution to be exaggerated; or to prevent the inconveniences of a check on the financial operations of the persons who distribute the funds."

The editor proceeds to detail the results deducible from the present Reports:

6. "Dividing the total deaths by the number treated, (2) exhibits the mortality of the cases. Example: Canterbury Infirmary, 1826-35, in-patients treated, 4254; died, 198; and 198 divided by 4254 is .0465, or 4.65 per cent.

7. Dividing the yearly deaths by the average number under treatment gives the annual rate of mortality; dividing the result by 10 presents the rate of mortality in 36.5 days. Thus the deaths out of a given number, in a given time, can be compared at different institutions; the mortality of the sick may be compared with that of the total population: and, moreover, 36.5 days is an approximation to the mean duration of severe cases. Example—Canterbury Infirmary, 1826-35, the average number of patients under treatment was 54.6, the annual deaths 19.8; and 19.8 divided by 54.6 gives, for the annual rate of mortality, .363, or 36.3 per cent., and a mortality, in 36.5 days, of 3.63 per cent.

8. The average nnmber under treatment (5) multiplied by 365¼, and divided by the total number treated, (2) presents the mean term of treatment in days. Example—the Canterbury observations as above. The average number under treatment, 54.6, multiplied by 365¼, is equal to 199, 426 days of treatment, which divided by 4254, the number treated, shows that the mean term of treatment was 47 days.

In the present hospital-books, the age, sex, residence, and occupation are generally entered. The remarkable results presented by the mortality of attacks at different ages have been pointed out in other parts of this Almanack. The sexes and occupations, as well as the ages of the patients admitted, cured, and dying, should be inserted in the reports.

From the numbers *admitted* at each age,—for instance, 20—30, and 40—50; and enumerations of the numbers *under treatment* at the same intervals of age, it may be ascertained to what extent the term of treatment varies with the age of the patients. If for 100 treated in the year, between the ages of 20—30, ten remained constantly under treatment, and for 100 admitted between the ages 40—50 fifteen remained on an average, it would follow that the patients aged 20—30 remained 36½ days, the patients aged 40—50 remained as long again, or 54 3-4 days. The ages of the patients admitted in quinquennial periods of life should therefore be published; and, in addition to this, the mean number at the same ages constantly under treatment deduced from twelve annual enumerations made on the first day of every month."

From a review of the preceding observations, it is obvious that the principal addition to reports of cases recommended by the Editor, is the stating with accuracy the duration of the disease—the number of days that the patient has been ill before admission—the number of days that he continues under treatment. Accuracy is above all things to be desired—and next to accuracy comes method. It is clear that the time is arriving when the medical officers of hospitals will find their best interest, in rendering the facts which occur in those institutions as extensively useful as possible to the profession. System will enable them to offer those facts in the best form at the least trouble. It is on this account that we have made the preceding extracts from the useful little work which lies before us. Our readers know how much we have advocated the publication of statistical results—how unceasingly we have encouraged the study and the diffusion of facts both in the form of particulars and of generalizations. We would fain hope that our endeavors "to guide and to improve the public taste" have not been altogether useless, and that our influence, temperate and rational in its objects and its character, is more permanent and more beneficial than that of mere political brawlers.—*Med. Chir. Rev.*—*Select Med. Library.*

—

19. *Use of the Solid Nitrate of Silver for the Gonorrhœa of Women.*—There is a very warm recommendation of the nitrate of silver, by Dr. Hannay, of Anderson's University, Glasgow, in the Medical Gazette.*

Gonorrhœa in women is generally said to be a very curable complaint. We cordially agree with Dr. Hannay in denying in toto that it is so. Nay, we will go farther than he does; for while he admits that it is "much more so than in men" we believe that, on the whole, it is less so. The acute symptoms are pretty readily removed, it is true; but a chronic discharge is generally set up, and when set up, is almost always obstinate.

The following is Dr. Hannay's mode of applying the caustic;—

"I introduce a stick of nitrate of silver into a quill, and tie a thread firmly round the lower part of the quill, to fasten the caustic, which I leave projecting beyond the quill about half an inch. I generally smear the quill with a little lard, and introduce the nitras argenti up to the os tincæ, or as far as it can be made to ascend in the vagina. I then deliberately and slowly withdraw it, turning it round so as to bring it in as extensive contact as possible with the lining membrane of the vagina. I may add, that by accident the nitrate of silver has more than once broken in the vagina, and could not be found. It caused me much alarm and anxiety at first, but after the following case I was not so affected; and though I would carefully avoid it, I now regard the occurrence as of very little importance.

The late Mr. John Herbertson, who acted as my assistant in the hospital, came in breathless haste, and in the utmost state of alarm, to call me to his assistance to extract a piece of the nitrate of silver from the vagina of a woman, who, having previously been in the hospital, and cured by the use of the remedy in question, came to Mr. H. to have it again applied. On repairing to the house we searched in vain for the caustic, but had abundant proofs that it had dissolved in the vagina. The quantity he asserted to be above two drachms. She suffered little or no pain, and a perfect cure was straightway accomplished."

It would appear that this has become a sort of party question in

---

* Medical Gazette, May 6.

Glasgow. Some persons there have denounced the practice as barbarous and dangerous. On these Dr. Hannay, and some friends of his who correspond with him, are exceedingly severe; and all parties would appear to have been infected with the subject, and to be exceedingly *caustic* towards each other. To evince Dr. Hannay's confidence, it is only necessary to quote such a passage as this:—

"I fearlessly give it out as an infallible and safe remedy for this disease, without any one drawback but the vain fears of persons of no experience, or of such as are determined to oppose it. I have now employed it in above 300 cases with unvarying success, and shall continue to use it.—*Med. Chir. Rev.*—*Ibid.*

---

20. *Case of Recovery from the insensibility of Intoxication, by the performance of Tracheotomy.* By S. Sampson, Esq., of the Salisbury Infirmary.

*Case.*—"Abraham Harris, aged 31, was brought to my house on the 31st of March last, in a state of complete insensibility from intoxication, the pupils being largely dilated, the breathing stertorous, and all voluntary motion having been lost for at least four hours before I saw him. The account given by those who came with him was, that he had attended a convivial meeting in the course of the day, at which he had drunk freely both of beer and brandy; his companions admitted that he had taken more than a pint of the latter, but it has since been ascertained that his glass was repeatedly filled up, without his knowledge, with white brandy, instead of water, so that it is impossible to calculate what quantity of spirit he had actually taken.

I immediately used the stomach pump, and drew off between three and four pints of fluid, a great part of which appeared to consist of brandy; after which tepid water with ipecacuanha diffused in it was several times injected into the stomach, and after a while withdrawn again, with a view to excite vomiting, and thus rouse the energies of the brain. Finding, however, that these means failed, a strong solution of salt in water, and afterwards the sulphate of zinc, were repeatedly tried, without any better result; but he became, if possible, more comatose, the countenance turgid, breathing more and more difficult; the pulse grew fainter, and was at last scarcely perceptible; at the same time the whole surface of the body was cold and clammy, and he was insensible to every kind of stimulus. As he was some miles from his home, I had him removed to the Infirmary, and called a consultation of the other medical attendants, who arrived in the course of half an hour; but as, in addition to the above symptoms, he had lost the power of swallowing, and every appearance indicated the rapid approach of death, nothing was ordered for him but a turpentine injection, there being no ground to justify a reasonable hope of recovery.

At this period it occurred to me, whilst standing by his bed-side, that the comatose state in which he lay might not arise from apoplexy, but from torpor of the brain, in consequence of that organ being supplied with blood not duly oxygenated; for the shrill tone and extreme difficulty of respiration showed the existence of collapse of the glottis, and imperfect transmission of air into the lungs, which might be accounted for by a paralyzed state of the eighth pair of nerves and recurrent branches.

With this view of the case I again appealed to my colleagues, and strongly urged that a trial should be given to the operation of tracheotomy; for I could not but hope that if mechanical respiration were carried on for a time, the blood might retain its proper stimulant properties, and restore the energies of the brain and nervous system. Upon their consenting to give him this chance, the operation was performed, without loss of time, by Mr. Andrews, under whose care, as surgeon for the week, the patient was now placed.

The trachea was no sooner opened than the distension of the veins about the head and neck subsided, the violent efforts of the extra-respiratory muscles ceased, and in about half an hour regular and easy respiration through the wound was completely established; at the same time the pupils became slightly sensible to the stimulus of light, and the pulse returned to the wrist. The immediate result of the operation being thus far satisfactory, nothing remained to be done but to give directions for the frequent removal of the mucus which appeared at the wound, and to keep the surfaces of the incision asunder until the integuments and muscular layers had become agglutinated to each other; this latter object was affected by means of a piece of strong spring wire, with a bow at each end of it, which, being introduced in a bent state, was allowed to expand, and the opening in the trachea was thus prevented from being covered by the muscles, even during the efforts of deglutition.

He continued perfectly quiet during the night, but had no return of consciousness until the following morning, when he gave us to understand, by signs, that he suffered from headache and soreness at the pit of the stomach; there was a tendency to sickness, and the tongue was coated with a peculiar whiteness, as if rubbed over with chalk. Moderate purgitives, followed by mild alkaline medicines, soon removed these symptoms, and a few leeches were applied to the throat, for the purpose of checking too high a degree of inflammation; after which no further treatment was required; but the wound being healed in about three weeks, he was discharged cured, and has continued up to this time in the enjoyment of perfect health."

We think there can be little doubt that the patient was dying of asphyxia, the result of the depressing influence of alcohol upon the brain and nerves. The opening made in the trachea facilitated respiration, by establishing an aperture, not muscular and regulated by nervous influence like the glotis. In such a case, were the symptoms still more urgent, and tracheotomy ineffectual, artificial respiration should, of course, be kept up. The effects of alcohol are transitory, and the view with which Mr. Sampson advised the operation is perfectly philosophical, and was, happily, successful.—*Ibid,*

---

21. *Case of Recovery from the influence of Opium by Artificial Respiration.* By C. Irving Smith, Esq., of the Mary-le-bone Infirmary.

This case may be considered a valuable pendent to the one immediately preceding. It, too, is contained in the last volume of the Medico-Chirurgical Transactions.

*Case.*—Jane H., aged 25, a stout young woman, was admitted into the St. Mary-le-bone Infirmary, on the 20th of July, 1828.

"At six o'clock in the morning, she was observed by one who slept in the same room, to swallow something from a cup, the remains of which was opium. She shortly after became insensible. At 10 A. M. she was brought in. At this time her extremities were cold and livid; the lips and face of a dark lead colour, the pulse fluttering, and scarcely perceptible at the wrist. Respiring three or four times in a minute, with sighing.

The stomach pump was applied, and that organ was carefully washed out, first with water, then with dilute acetic acid; after which, small quantities of ammonia were injected, combined with brandy. The spasm of the œsophagus was so great, on attempting to introduce the tube of the stomach pump, as nearly to destroy it.

The patient was now (11 A. M.) evidently worse than at the time of her admission; so much so, that many doubted if she still lived. The hair was next entirely cut off from the whole head, and the head placed over the edge of the bed, several buckets of cold water were poured in rapid succession over it; the nostrils being at the same time stimulated with volatile alkali. The pulse now intermitted much at the wrist, at one time being 70 or 80 beats in the minute, and the next sinking as low as 7 or 8. Respiration had now nearly ceased.

The scalp was now rubbed with the liq. ammoniæ until the entire of it was vesicated.

11½ A. M., it was determined, as the only remaining chance, to try the effects of artificial respiration; there being at this time no pulse whatever at the wrist, and only a slight irregular action at the heart, indicative that life was not quite extinct.

The mouth and one nostril being closed, a pair of common bellows was applied to the other nostril, and the chest was in that way inflated, and alternately emptied by pressure on the chest and sides. This was continued for an hour, without intermission, at which time the heart seemed to be rallying, but if left to itself, it rapidly sunk again. An ounce of the ol. terebinth was injected into the rectum. Bottles of hot water were applied to the chest, and sinapisms to the feet and legs.

The same treatment was continued till 2 P. M., with only very slight intermission. She was at this time deemed so evidently rallying, that it was deemed safe to leave her.

She was again seen at 3 P. M., when she was found relapsing into her former state. Recourse was again immediately had to artificial respiration, which was continued till 5 P. M., at which time it was suspended, on account of the pulse having become regular, and her having made some slight attempts to move; and showing evidence of pain on being pinched.

At 12 P. M., she became slightly sensible, and swallowed a little tea. She became eventually well, and has since married.

It was curious to see the effects of artificial respiration in this case. The livid color of the face and extremities, rapidly giving way to a more florid hue, on every inflation, and again as rapidly returning to its original color, on being left for a few minutes."—*Med. Chir. Trans.*

